# Growth of Low-Defect WSe_2_ Film via High-Purity van der Waals Crystal Precursor

**DOI:** 10.1021/acsnano.5c21076

**Published:** 2026-03-16

**Authors:** Hang Liu, Ni Yang, Jiacheng Min, Mykola Telychko, Caisheng Tang, Yi Wan, Chuanqi Zhang, Wanying Li, Lin-Yun Huang, Chengdong Yao, Hoyeon Jeon, Jiahao Liu, Zhengwei Zhang, Xiangdong Yang, George Harrison, Zhongzhe Liu, Tianchao Guo, Jing-Kai Huang, Shadi Fatayer, Kaimin Shih, Song Liu, Thomas D. Anthopoulos, Kian Ping Loh, Lain-Jong Li, Xu Lu

**Affiliations:** † Division of Physical Science and Engineering (PSE), King Abdullah University of Science and Technology (KAUST), Thuwal, 23955-6900, Kingdom of Saudi Arabia; ‡ Department of Mechanical Engineering, 25809 The University of Hong Kong, Hong Kong, China; § Department of Civil Engineering, 25809 The University of Hong Kong, Hong Kong, China; ∥ Center for Nanophase Materials Sciences, 6146 Oak Ridge National Laboratory, Oak Ridge, Tennessee 37831, United States; ⊥ Department of Applied Physics, 26680 The Hong Kong Polytechnic University, Hung Hom, Kowloon, Hong Kong, China; # Department of Chemistry, 37580 National University of Singapore, Singapore 117543, Singapore; ¶ Hunan Key Laboratory of Nanophotonics and Devices, School of Physics and Electronics, 12570 Central South University, Changsha 410083, China; □ Institute of Micro/Nano Materials and Devices, Ningbo University of Technology, Ningbo 315211, China; ○ Department of Systems Engineering, 53025 City University of Hong Kong, Hong Kong, China; △ Hunan Key Laboratory of Two-Dimensional Materials and State Key Laboratory for Chemo/Biosensing and Chemometrics, College of Chemistry and Chemical Engineering, Hunan University, Changsha 410082, China; ▲ Henry Royce Institute and Photon Science Institute, Department of Electrical and Electronic Engineering, The University of Manchester, Oxford Road, Manchester, M13 9PL, United Kingdom

**Keywords:** two-dimensional semiconductors, P-type, low-defect, WSe_2_, field-effect transistors, scanning tunnelling microscope

## Abstract

Two-dimensional (2D) semiconducting transition metal dichalcogenides (TMDs) exhibit exceptional electrical and optical properties, empowering their promising prospects for future nanoelectronics. Despite major advances in n-type 2D semiconductors, the field has yet to synthesize high-mobility p-type 2D TMDs, in particular WSe_2_, and systematically query the influence of defects. In this study, we unveil the pivotal role of substitutional impurity defects vis-à-vis the precursor used and growth method employed in defining the quality of 2D p-type WSe_**2**_. Density functional theory calculations suggest the adverse effect of Fe-, Co-, Ni- and Si-substituted W impurity defects on the mobility of WSe_2_, whereas defects such as O-, S-substituted Se and Mo-substituted W pose negligible impact. Guided by the theory, we pinpoint van der Waals (vdW) crystals, commonly used in mechanical exfoliation, as the optimal precursor, and develop a facile vdW crystal physical vapor deposition (PVD) method to grow high-purity monolayer 2D WSe_**2**_ film (VPVD-WSe_2_) that is continuous across a centimeter scale. A suite of spectroscopies confirms the markedly reduced defect density of the as-synthesized WSe_**2**_ compared to those by typical chemical vapor deposition methods, and by PVD with commercial or hydrothermal precursors. Scanning tunneling microscopy further evidence the ultralow substitutional impurity defect density of VPVD-WSe_2_, greatly outperforming the control samples and approaching the mechanically exfoliated counterparts. The VPVD-WSe_**2**_ based field-effect transistors exhibit notable electrical performance with record-high field-effect hole mobility up to 112 cm^2^ V^–1^s^–1^ at room temperature, exceeding the best-reported monolayer WSe_2_ synthesized by chemical vapor deposition and rivaling the mechanically exfoliated 2D WSe_2_ flakes.

## Graphical abstract

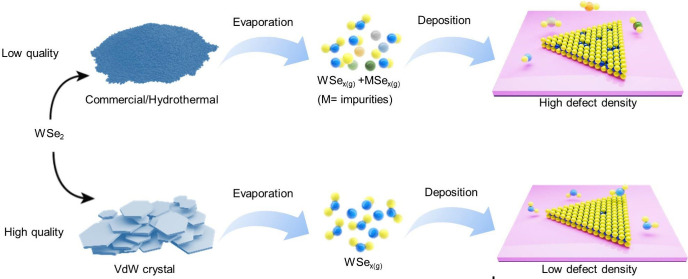

Two-dimensional (2D) semiconducting transition metal dichalcogenides (TMDs) demonstrate excellent electrical properties, such as high carrier mobility, tunable bandgap, and superb electrostatic control, rendering them promising candidates to extend Moore’s Law for next-generation electronics.^1–3^ Among 2D TMDs, WSe_2_ stands out owing to the ambipolar characteristics, empowering its versatility across multiple electrical and optoelectronic applications with complementary functions.^3–7^ Nevertheless, the mobility of scalable 2D p-type WSe_2_ monolayers, prevalently fabricated by the chemical vapor deposition (CVD) method, still lags behind the n-type 2D semiconductors^8,9^ and falls short of the theoretical limit.^10–13^

Point defects have been widely believed to constrain the electrical properties of 2D TMDs.^14–18^ However, controversy persists over the influence of defects on the mobility. For example, charge trapping Se vacancies were regarded as the primary cause of the low WSe_2_ mobility.^19^ This notion was then challenged by scanning tunneling microscopy (STM), revealing that oxygen tends to overwhelmingly passivate Se vacancies to form oxygen-substituted Se (O_Se_) defects in WSe_2_.^20–22^ Subsequent theoretical studies suggested that such defects do not introduce in-gap states and may not affect the WSe_2_ mobility;^22–25^ meanwhile, Xiao et al. inferred that filling chalcogen vacancies with oxygen should in general improve the mobility of 2D TMDs.^26^ The field was further puzzled by recently discovered impurity-substituted W (Im_W_) defects,^21^ albeit lacking in-depth mechanistic insights. It is therefore highly desired, but not yet realized, to explore how specific types of defects jeopardize the mobility of 2D WSe_2_.

Physical vapor deposition (PVD), an emerging technique for crafting high-quality 2D thin materials by directly sublimating the precursors, may well serve this purpose.^4,27–31^ The commercial PVD precursors usually manifest a maximum purity of about 99.8%.^4,29^ At high temperatures, the impurities are volatilized together with the precursors, leading to the formation of substitutional impurity defects. This allows us to explicitly probe the influence of different types of defects on 2D WSe_2_ mobility.

Here, we ascertain the role of various defects on the mobility of WSe_2_, underscoring the importance of controlling the substitutional impurity defects for synthesizing high-mobility 2D materials. Density function theory (DFT) calculations suggest that the in-gap states introduced by Fe-, Co-, and Ni-substituted impurity defects impose an adverse effect on the charge mobility of WSe_2_. On the contrary, defects such as O-, S-substituted Se and Mo-substituted W exert no such impact. The theoretical prediction guides us to develop a van der Waals (vdW) crystal PVD (VPVD) method for the growth of high-purity continuous monolayer p-type WSe_2_ film at centimeter scale. The as-synthesized WSe_2_ manifests a drastically reduced substitutional impurity defect density of 8 × 10^10^ cm^–2^, as evidenced by a suite of microscopies and spectroscopies. We then showcase our VPVD-WSe_2_ on field-effect transistors (FETs). Consistent with the ultralow substitutional impurity defect density, the VPVD-WSe_2_ based devices exhibit remarkable electrical performance with a hole mobility of 112 cm^2^V^–1^s^–1^ at room temperature, among the highest for WSe_2_ monolayers grown by vapor deposition.

## Selection and Characterizations of Precursors

The theoretical predictions (Figure S1 and discussions) motivated us to manipulate substitutional impurity defects for high-mobility WSe_2_. We posit that this can be realized using the PVD technique since the precursors are sublimated and redeposited, and the defect density of the yielded products may be highly dependent on the quality of precursors (Figure 1a). To test this postulation, we adopted three WSe_2_ precursors with distinct grades – that is, commercially available,^27–31^ hydrothermally synthesized,^32^ and chemical vapor transportation (CVT)-grown vdW crystal sources (Figure S2a; details in the Methods). The commercial and hydrothermal samples appeared gray and dark (Figure S2b, c), while the vdW crystal sample possessed a highly glossy crystalline appearance (Figure S 2d). Scanning electron microscopy (SEM) confirmed the morphological disparity among the three precursors: the commercial and hydrothermal powders exhibited irregularly flaky and nanoflower-like morphologies, respectively (Figure S2e, f), whereas the vdW crystal presented a highly crystalline hexagonal structure (Figure S2g).

**Figure 1:**
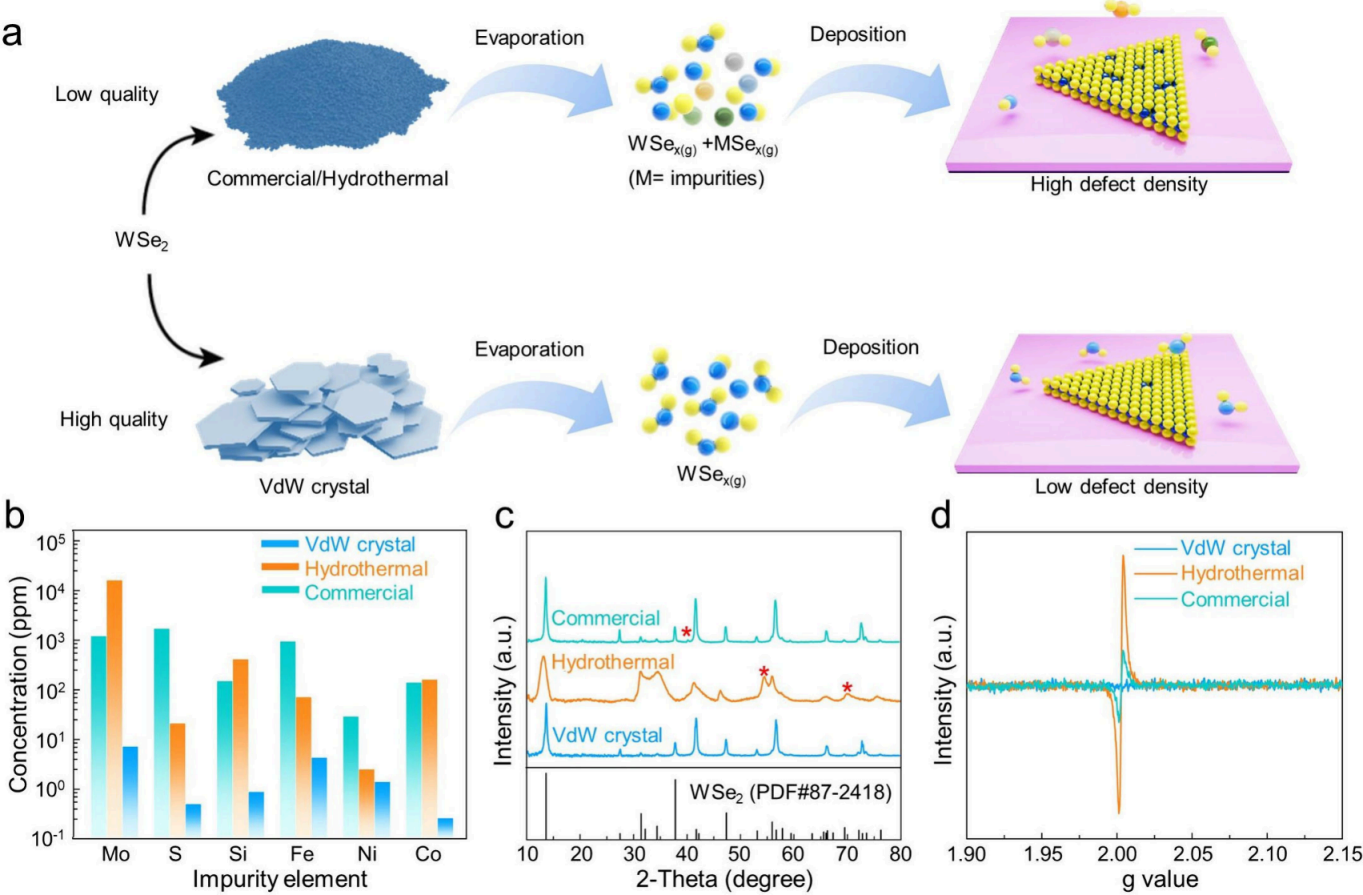
Schematic illustration of WSe_2_ monolayer growth and characterization of precursors. (a) PVD growth of WSe_2_ monolayers using precursors with distinct grades. (b) GD-MS, (c) XRD and (d) ESR spectra of commercial, hydrothermal and vdW crystal WSe_2_ precursors.

We then conducted elemental analysis to quantitatively differentiate the grades of the precursors. Glow discharge mass spectrometry (GD-MS) revealed the presence of impurities including Mo, S, Si, Fe, Ni, and Co. Glow Discharge Mass Spectrometry (GD-MS) is an advanced analytical technique to assess the composition of solid samples, especially for identifying trace elements and impurities. By integrating glow discharge ionization with mass spectrometry, it enables comprehensive analysis of elemental composition.^33^ The concentrations of these impurities in CVT-grown vdW crystals were observed to be several orders of magnitude lower than those in the other two control samples (Figure 1b). We further carried out X-ray diffraction (XRD) through multiple samplings, which evidenced the existence of impurity phases (marked with asterisk) in both the commercial and hydrothermal precursors. Conversely, the XRD spectrum acquired on vdW crystals aligned well with the standard WSe_2_ pattern (PDF#87–2418), suggesting the negligible presence of impurity phases (Figure 1c and Figure S3). Additionally, electron spin resonance (ESR), capable of discerning the defects, indicated that both commercial and hydrothermal powders manifested a peak at g = 2.0, while no obvious peak was detected on vdW crystals (Figure 1d). This signifies the charge state linked to Se vacancies in the source materials, and the increased peak intensity implies a denser Se vacancy population.^34^ We then mechanically exfoliated WSe_2_ monolayers (ME-WSe_2_) from the CVT-grown bulk vdW crystal precursor to reflect its level of defect (Figure S4). The room temperature Raman spectroscopy of the ME-WSe_2_ monolayer showed strong E/A_1g_ and 2LA­(M) peaks at 250 and 259 cm^–1^, respectively, both of which can be assigned to WSe_2_ (Figure S4b). The room temperature Photoluminescence (PL) emission suggested a narrow fwhm of 21 nm (Figure S4c). These observations reaffirm the exceptional quality of the as-grown vdW crystals.

## Synthesis and Characterizations of High-Purity WSe_2_

The high quality of CVT-grown vdW crystals drove us to synthesize large-area, low-defect-density WSe_2_ monolayers through PVD. We chose the reverse-flow PVD system (Figure S5) to prevent the undesired crystal nucleation with precise monolayer thickness control,^27^ and realized the growth of centimeter-scale continuous monolayer WSe_2_ polycrystalline film on sapphire using the vdW crystal precursor (VPVD-WSe_2_, Figure 2a). The VPVD-WSe_2_ film was first assessed by optical microscopy, PL, and Raman intensity mapping over a 100 μm × 100 μm area, all of which indicated the exceptional uniformity of the sample (Figures 2b, c and Figure S6). Raman line scan with a step size of 0.02 cm also confirmed the homogeneity of the VPVD-WSe_2_ monolayer film: the WSe_2_ characteristic peak intensity remained largely unchanged across a distance of 1 cm (Figure 2d). Atomic force microscopy (AFM) determined the thickness of the VPVD-WSe_2_ film to be 0.75 nm (Figure 2e).

**Figure 2:**
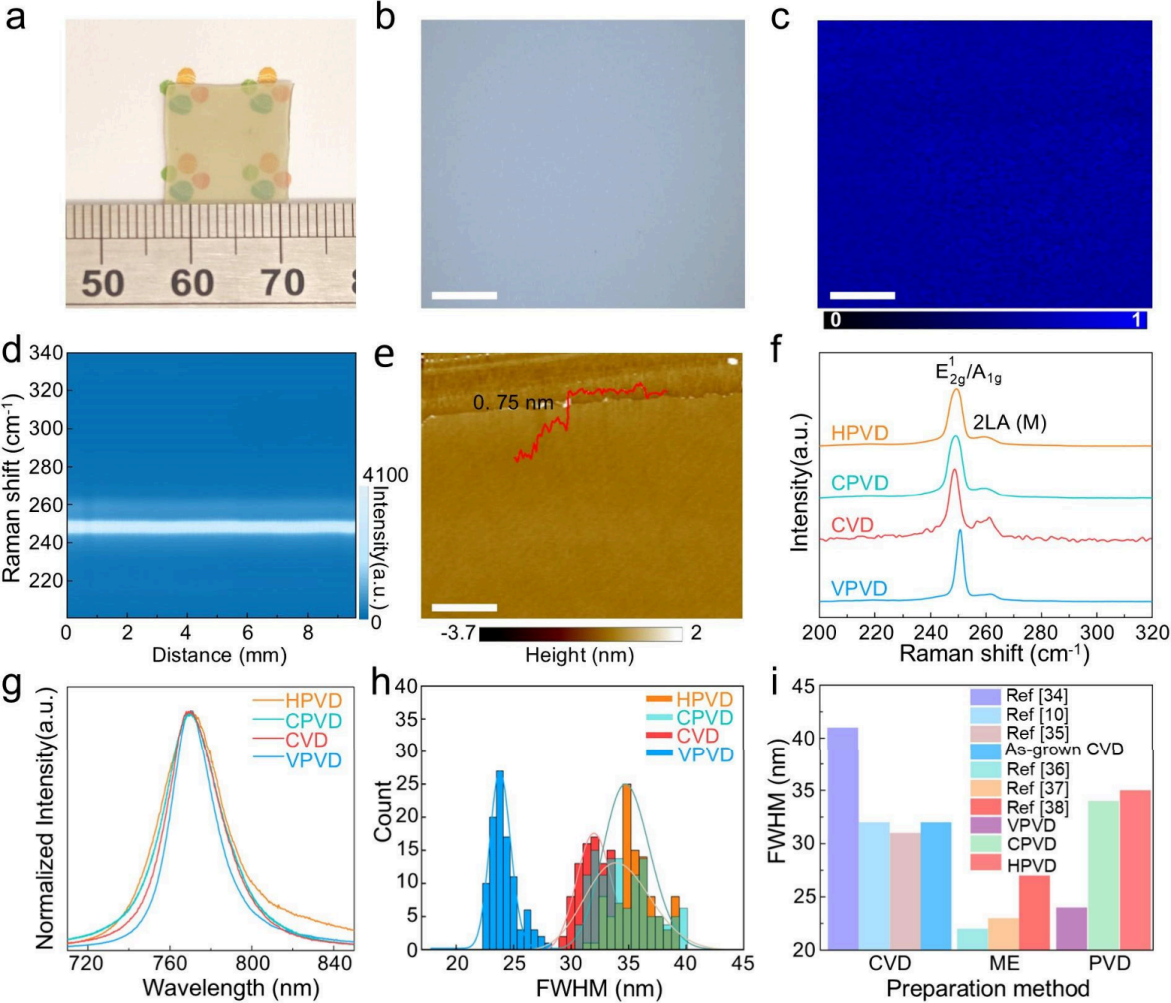
Characterizations of WSe_2_ monolayers. (a) Photograph, (b) optical microscopy and (c) PL mapping of a continuous monolayer VPVD-WSe_2_ film (scale bar, 20 μm). (d) Color-coded Raman peak profiles across a distance of 1 cm on a VPVD-WSe_2_ film. (e) AFM image of the VPVD-WSe_2_ monolayer film grown on sapphire substrate (scale bar, 3 μm). (f) Raman, (g) selected PL spectra and (h) statistical distribution of PL FWMH for CPVD-WSe_2_, HPVD-WSe_2_, CVD-WSe_2_ and VPVD-WSe_2_ monolayers on SiO_2_/Si. (i) PL fwhm of PVD-grown, CVD-grown^10,35,36^ and ME-WSe_2_ ^37–39^ reported in the literature.

We further examined the quality of the VPVD-WSe_2_ flakes, benchmarked by commercial (CPVD-WSe_2_), hydrothermal (HPVD-WSe_2_) and conventional CVD (CVD-WSe_2_) synthesized WSe_2_. All the samples were monolayers as confirmed by AFM (Figure S7). Both the Raman and PL spectra showed WSe_2_ peaks with considerably narrower fwhm on VPVD-WSe_2_ than the other three control samples (Figure 2f, g). Moreover, Raman and PL mappings revealed the nonuniform WSe_2_ peak intensity distributions on CPVD-WSe_2_, HPVD-WSe_2_ and CVD-WSe_2_ in sharp contrast to VPVD-WSe_2_ (Figure S8e–l). We then assessed the defect levels in the as-prepared WSe_2_ monolayers by quantitatively analyzing one hundred room temperature PL spectra that were collected from multiple flakes. The statistics indicate an average fwhm of 24 nm for VPVD-WSe_2_, significantly lower than that of CPVD-WSe_2_ (34 nm), HPVD-WSe_2_ (35 nm), CVD-WSe_2_ (32 nm) and other up-to-date CVD-grown WSe_2_, and approaching the ME-WSe_2_ (Figure 2h, i). These findings affirm the strong dependence of PVD-grown WSe_2_ on the quality of their precursors, and exhibit the notably high quality of VPVD-WSe_2_ with extremely low defect density.

We then sought to evaluate the quality of the as-prepared WSe_2_ at atomic level. Scanning transmission electron microscopy (STEM) scans over large (30 nm × 30 nm) and small (5 × 5 nm^2^) areas were first performed. The STEM images in Figures S9 and 10 showed reduced cluster defects in VPVD-WSe_2_ monolayers, while CPVD-WSe_2_ and HPVD-WSe_2_ manifested abundant Se defects, including cluster defects. We further used scanning tunneling microscopy (STM) to accurately probe the type and density of defects, which is beyond the capability of STEM. To avoid cross-contamination during the transfer process, each WSe_2_ monolayers were directly grown on highly oriented pyrolytic graphite (HOPG) substrates. HOPG substrates were employed as the growth substrate in light of its high conductivity, which allows for enhanced STM imaging. The defect levels in the as-grown WSe_2_ monolayers on HOPG were comparable to those grown on other substrates (for example, SiO_2_/Si and sapphire), as examined by the room-temperature PL measurements (Figure 2g and Figure S11). The 50 nm × 50 nm STM images evidenced a substantially denser population of structural defects on CVD-WSe_2_, CPVD-WSe_2_ and HPVD-WSe_2_ compared to VPVD-WSe_2_, in line with previous spectroscopic observations (Figure 3a-c and Figure S12). The structural defects can be further classified into three types – that is, oxygen-substituted bottom Se (O_Se_ bottom), oxygen-substituted top Se (O_Se_ top) and Im_W_, as featured in the magnified STM images and d*I*/d*V* spectra (Figure 3d and Figure S13).^20,40^ Other defects, such as S_Se_, exhibited a minimal presence (Figure 3a-c and Figure S12), thereby posing negligible impact on hole mobility, consistent with our theoretical predictions. Statistical analysis (Figure 3e and Supplementary Table 2) unveiled an O_Se(top)_ defect density of 1.8 × 10^12^ cm^–2^ on VPVD-WSe_2_, 1 order of magnitude lower than that of CPVD-WSe_2_ (2.8 × 10^13^ cm^–2^), and of the same order of magnitude as HPVD (4.4 × 10^12^ cm^–2^) and CVD-WSe_2_ (4.6 × 10^12^ cm^–2^). The O_Se(bottom)_ defect density of 1.76 × 10^12^ cm^–2^ on VPVD-WSe_2_, 1 order of magnitude lower than that of CPVD-WSe_2_ (2.17 × 10^13^ cm^–2^), and of the same order of magnitude as HPVD (3.4 × 10^12^ cm^–2^) and CVD-WSe_2_ (1.96 × 10^12^ cm^–2^). Likewise, the Im_W_ defect density of VPVD-WSe_2_ (8 × 10^10^ cm^–2^) was 2 orders of magnitude lower than that of CPVD-WSe_2_ (4.12 × 10^12^ cm^–2^), HPVD- WSe_2_ (5.6 × 10^12^ cm^–2^) and 1 order of magnitude lower than that of CVD-WSe_2_ (8 × 10^11^ cm^–2^). To rigorously evaluate the overall defect density, STM scans were conducted on eight additional, randomly selected 50 nm × 50 nm areas of multiple VPVD-WSe_2_ monolayers (Figure S14a–h). The average defect density of VPVD-WSe_2_, including O_Se_ bottom, O_Se_ top and Im_W_, were calculated to be (4 ± 0.5) × 10^12^ cm^–2^ (Figure S14i), comparable to those synthesized by metal–organic CVD (MOCVD) and mechanical exfoliation that typically yield low-defect-density WSe_2_ (Figure 3f).^20,21,40^ The WSe_2_ mechanically exfoliated from our bulk crystals exhibited 7.5 × 10^10^ cm^–2^ O_Se_ defect density and 3.75 × 10^10^ cm^–2^ Im_W_ defect density (Figure S15). The difference in the defect densities between the VPVD-WSe_2_ and ME-WSe_2_ is likely caused by the VPVD process. High-resolution X-ray photoelectron spectroscopy (XPS) was then performed to identify the impurity elements. Fe, Co, and Ni 2p peaks were exclusively observed on CPVD-WSe_2_, whereas no such peaks were detected on VPVD-WSe_2_ (Figure S16). The obvious presence of Fe, Co, and Ni impurities in CPVD-WSe_2_ and their low concentrations in VPVD-WSe_2_ agree well with GD-MS results of the corresponding precursors (Figure 1b). The VPVD-WSe_2_ also matches with the theory-predicted defect profile of a high-quality WSe_2_ (Figure S1), primed for ensuing electrical measurements.

**Figure 3:**
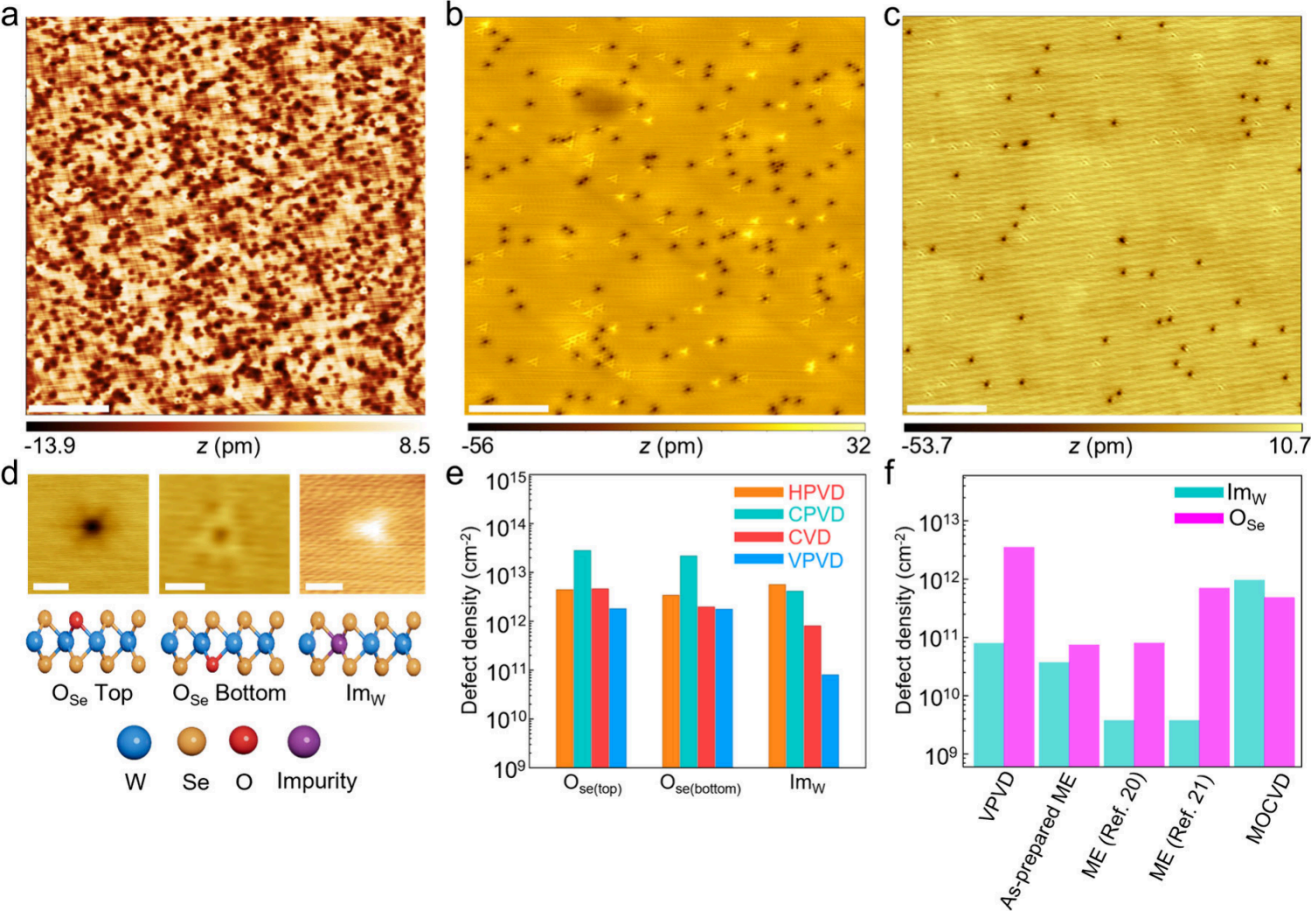
Defect analysis by STM. (a–c) STM images (scale bar, 10 nm) of (a) CPVD-WSe_2_ (bias voltage (V) = 2 V, current (I) = 40 pA), (b) CVD-WSe_2_ (V = 1.3 V, I = 8 pA) and (c) VPVD-WSe_2_ (V = 2.2 V, I = 10 pA). (d) Magnified STM images of WSe_2_ monolayers showing O_se_ top, O_se_ bottom and Im_W_ defects (scale bar, 1 nm), and the corresponding side-view atomic structures. (e) Comparison of O_se_ and Im_W_ defect densities in HPVD-, CPVD-, CVD- and VPVD-WSe_2_. (f) Comparison of O_se_ and Im_W_ defect densities in VPVD-, ME- ^20,21^ and MOCVD-WSe_2_.^40^

## Electrical Performance of WSe_2_ Based FETs

The theoretical calculations and characterizations suggest that the mobility of our VPVD-WSe_2_ may be exceptional. To test this hypothesis, we fabricated VPVD-WSe_2_ based FETs by transferring the centimeter-scale continuous monolayer WSe_2_ film onto a SiO_2_/Si substrate. Figure 4a showed the configuration of a FET with Pt/Au as contact electrodes (20 nm Pt followed by 20 nm Au) and 270 nm SiO_2_ as gate dielectrics. A 0.8 mm × 0.7 mm optical image illustrated the representative array of back-gated FET devices and the zoomed-in image showed a typical FET with a channel length/width (L_ch_/W) of 3 μm/12 μm (Figure 4b). The output curves (drain-to-source current, *I*_*d*_, versus drain-to-source voltage, *V*_*ds*_) of a typical VPVD-WSe_2_ FET showed linear characteristics (Figure 4c). The transfer curves (*I*_*d*_ versus gate-to-source voltage, *V*_*gs*_) of one hundred sixty-five randomly selected VPVD-WSe_2_ FETs at *V*_*ds*_ = −1 V exhibited a p-type semiconductor behavior (Figure 4d), with a maximum hole field-effect mobility (μ_*FE*_) of 112 cm^2^V^–1^s^–1^ at a stable on/off ratio (*I*_*on*_/*I*_*off*_) of 1.4 × 10^8^ at room temperature (Figure 4e). The temperature-dependent transfer curve of a typical VPVD-WSe_2_ FET showed that the mobility increased with decreasing temperature, achieving a hole mobility of 175 cm^2^V^–1^s^–1^ at 15 K (Figure S17). Statistics of the tested FETs signified the notable electrical properties of the VPVD-WSe_2_ film – that is, a hole μ_*FE*_ of 69 ± 11.3 cm^2^V^–1^s^–1^, an *I*_*on*_/*I*_*off*_ of (3.4 ± 2.0) × 10^8^, and an on-state current (*I*_*on*_) of 9 ± 3.1 μA/μm (Figure 4f and Figure S18). We then benchmarked the electrical performance of VPVD-WSe_2_ against CPVD-WSe_2_, HPVD-WSe_2_ and CVD-WSe_2_. The average hole μ_*FE*_ of HPVD-WSe_2_, CPVD-WSe_2_ and CVD-WSe_2_ based FETs were measured to be 0.31, 14, and 29 cm^2^V^–1^s^–1^, respectively, significantly lower than that of VPVD-WSe_2_ (Figure 4g and Figure S19). The contact resistance values of CPVD-WSe_2_, HPVD-WSe_2_, CVD-WSe_2_ and VPVD-WSe_2_ FETs with Pt contacts were extracted by the transfer length method (TLM) to exclude the effects of the electrodes. The contact resistances for the HPVD-WSe_2_, CPVD-WSe_2_, CVD-WSe_2_ and VPVD-WSe_2_ FETs were 20, 8.5, 13.9, and 8 kΩ μm at *n*_2D_ of 3.9 × 10^12^ cm^–2^, 3.7 × 10^12^ cm^–2^, 6.6 × 10^12^ cm^–2^ and 5.5 × 10^12^ cm^–2^, respectively (Figure S20). As a result, the enhanced mobility in VPVD-WSe_2_ can be predominantly attributed to the reduction in Im_W_ defect density. The poor electrical performance of the three control samples originates from their high-defect-density precursors, in line with DFT calculations. Moreover, the low defect density of the VPVD-WSe_2_ significantly reduced the hysteresis of the FET, indicating its low border traps and interface states (Figure S21).^21^ To eliminate the influence of the dielectric substrate and ensure a fair comparison, we have fabricated the CVD-WSe_2_ devices on the same 270 nm SiO_2_ gate dielectric. The statistical analysis (Figure S22) of the hysteresis revealed that the VPVD-WSe_2_ FETs still exhibit an order-of-magnitude lower hysteresis than the other types of WSe_2_ devices. Figure 4h and Supplementary Table 3 summarized the μ_*FE*_ of state-of-the-art 2D WSe_2_ based FETs as a function of *I*_*on*_/*I*_*off*_. Our VPVD-WSe_2_ not only represents the best among PVD- and CVD-grown WSe_2_, but also performs comparably with (or better than) the mechanically exfoliated single/multilayer WSe_2_, albeit with higher O_Se_ concentration (Figure 3f). These results again corroborate that the WSe_2_ mobility can be selectively mediated by certain types of substitutional impurity defects, consistent with the theoretical predictions.

**Figure 4:**
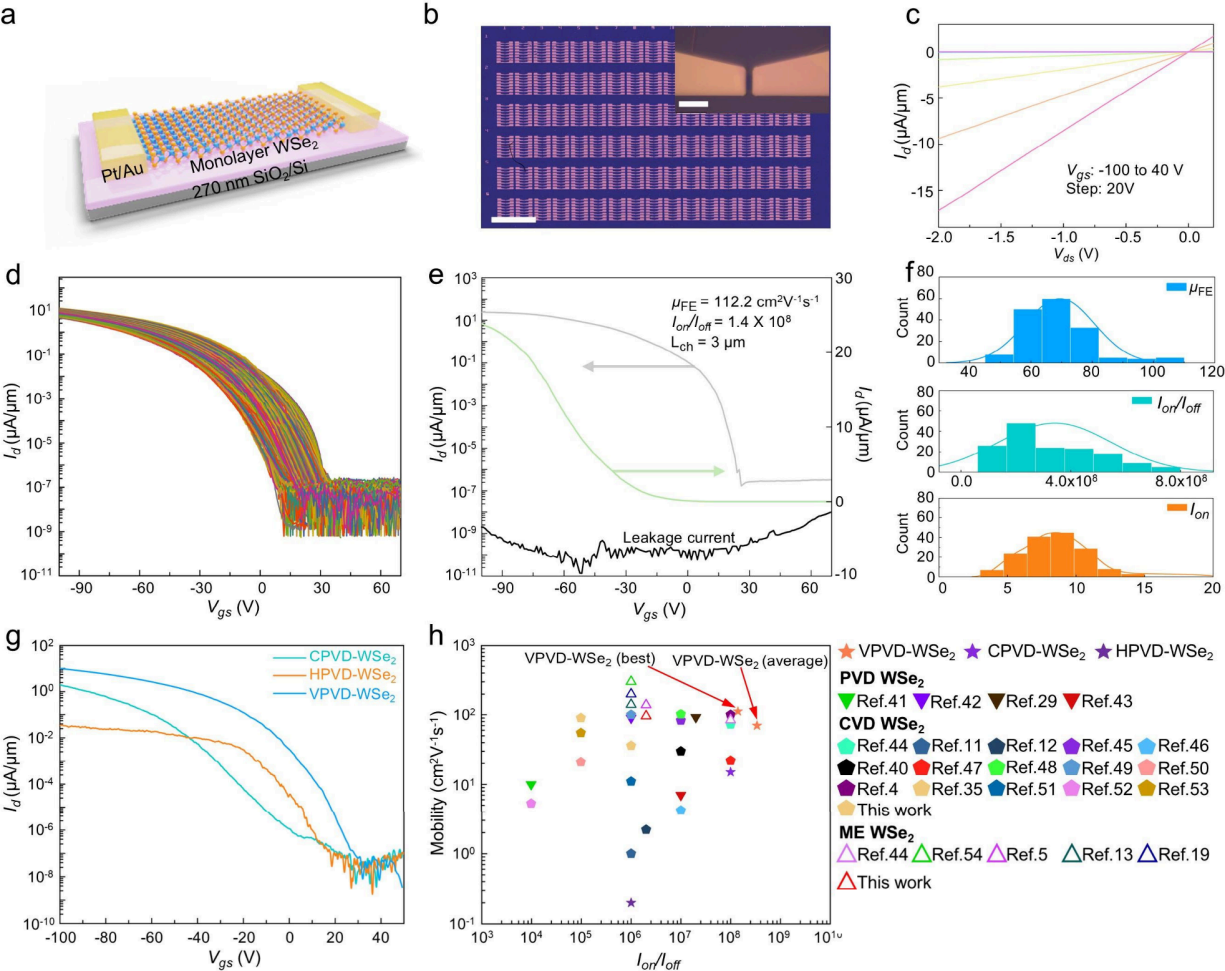
Electrical performance of WSe_2_ based FETs and benchmarks. (a) Schematic of a monolayer WSe_2_ based FET. (b) Optical microscopy image of the fabricated back-gated FET arrays (scale bar, 1 mm). Inset: zoomed-in view of a FET device (scale bar, 20 μm). (c) Output characteristics of a typical VPVD-WSe_2_ FET. (d) Transfer curves of one hundred sixty-five VPVD-WSe_2_ FETs at *V*_*ds*_ = −1 V. (e) Transfer curve for record-high hole μ_*FE*_ of 112 cm^2^V^–1^s^–1^ at an *I*_*on*_/*I*_*off*_ of 1.4 × 10^8^ and *V*_*ds*_ = −1 V. (f) Statistical distribution of hole μ_*FE*_, *I*_*on*_/*I*_*off*_ and *I*_*on*_ for the tested one hundred sixty-six VPVD-WSe_2_ FETs. (g) Transfer curves of CPVD-WSe_2_, HPVD-WSe_2_ and VPVD-WSe_2_ based FETs at *V*_*ds*_ = −1 V. (h) Summary of μ_*FE*_ in PVD,^29^ [,^41–43^ CVD,^4,11,12,35,40,44–53^ and ME synthesized WSe_2_ ^5,13,19,44,54^ with respect to *I*_*on*_/*I*_*off*_.

## Conclusion

To conclude, we demonstrated that the origin of low mobility in WSe_2_ can be traced to specific types of defects engendered in the crystal during growth, which can be further traced to the intrinsic impurities in the precursors used. Guided by theory, we developed a vdW crystal PVD method to minimize the defect density for centimeter-scale continuous monolayer WSe_2_ film. Our protocol establishes a new benchmark for vapor deposition-synthesized WSe_2_ FETs, and delivers comparable electrical performance to incumbent mechanical exfoliation techniques. In a broader context, this work is envisaged to extend to other 2D TMDs thin films and holds promise for the development of 2D complementary logic circuits, contingent upon further advancements in scalable production.

## Methods

### Preparation of Precursors

All chemicals and reagents, including the commercial WSe_2_ powder (99.8% purity), were purchased from Alfa Aesar and used as received without further purification. The hydrothermal WSe_2_ precursor was synthesized using the one-step solvothermal process.^32^ NaBH_4_, Se, and NaWO_4_ were first used as raw materials and mixed with 60 mL dimethylformamide (DMF). The solution was then mixed with 10 mL deionized water (Millipore, 18.2 MΩ cm), transferred into a Teflon liner loaded in a stainless-steel autoclave and kept at 200 °C for 48 h.

VdW crystals were synthesized by CVT using iodine as the transport agent. W (99.999%) and Se (99.999%) powders in a ratio of 1:2 were first mixed with iodine (99.999%), and sealed in an evacuated (10^–3^ Torr) quartz ampule. The ampule was then placed into a tube furnace and heated to 950 °C at a rate of 1 °C min^–1^ and held for 120 h. Given that large bulk crystals are difficult to volatilize, we avoided the long reaction times used in previous reports^20^ for large bulk crystal preparation. Instead, we chose a shorter reaction time (120 h) and utilized small-sized WSe_2_ vdW crystals (Figure S 1d) as the precursor in our PVD process. Finally, the ampule was cooled down to room temperature. For the synthesis of Fe doped WSe_2_ vdW crystals, Fe (99.999%) was used as dopant. For the synthesis of the 0.1, 0.5, and 2% Fe doped WSe_2_ bulk crystals, Fe, W and Se powders were mixed in molar ratio (0.001:1:2, 0.005:1:2 and 0.02:1:2, respectively).

### PVD Growth of WSe_2_ Monolayers

WSe_2_ monolayers and films were prepared by a reverse-flow PVD system. First, different types of precursors (2g) were loaded into the center of the quartz tube. A piece of sapphire (HOPG, SiO_2_/Si) substrate was then placed in the downstream heating zone with a distance of 12 cm. The growth process was performed under ambient pressure. Prior to heating, the quartz tube was purged with 2000 sccm Ar for 2 min. Subsequently, the tube furnace was ramped up to 1150 °C within 50 min with an Ar reverse flow of 50 sccm (from the substrate to the precursor). The Ar flow was switched to forward (from precursor to substrate) immediately after the temperature reached 1150 °C and kept for 1 min to grow single-crystal flakes and 6 min to synthesize the continuous film. Finally, the tube furnace was cooled down naturally. Noted that all the growth variables except for the precursor quality were kept the same during the process.

### CVD Growth of WSe_2_ Monolayers

WSe_2_ monolayers were grown by the conventional CVD method using WO_3_ (Sigma-Aldrich, 99.90%) and Se (Sigma-Aldrich, 99.99%) powders as the precursors. The center heating zone was heated to 900 °C, and the Se at the upstream was maintained at 270 °C. A piece of sapphire (HOPG, SiO_2_/Si) was placed at the downstream with an Ar/H_2_ flow at 30 Torr. The growth continued for 15 min, followed by natural cooling to room temperature.

### Material Characterization

The ESR spectra of precursors were recorded on a Bruker X-band A200 spectrometer (9.4 GHz, 77 K). The impurity elemental concentration analysis of precursors was conducted using GD-MS (Nu instruments, Astrum). The morphology and thickness of WSe_2_ monolayers were determined by AFM (Bruker Dimension Icon system). Raman and PL spectra were acquired using the WITec confocal spectrometer with a laser excitation wavelength of 532 nm. XRD was performed using the Bruker D8 Advance diffractometer with a Cu–Kα radiation. Different types of precursors, each weighing 2g, were used for the XRD analysis. The morphology of the precursors was examined by SEM (FEI Quanta 600). The atomic structure and defects were characterized by the FEI Titan Themis Z STEM equipped with a double Cs (spherical aberration) corrector operating at 80 kV. XPS was operated at an (Omicron) UHV system (10–10 mbar) with an Al K^α^ X-ray source (1486.6 eV) operating at 15 kV. High-resolution spectra were collected at a constant energy of 15 eV. All the types of WSe_2_ were measured under identical experimental conditions. Each spectrum was acquired over 9 h to improve the signal-to-noise ratio.

### STM and d*I*/d*V* Measurements

The STM measurements of the CPVD-WSe_2_, CVD-WSe_2_ and VPVD-WSe_2_ samples were performed under ultrahigh vacuum (UHV) conditions at a temperature of 77 K using a commercial Omicron LT-SPM instrument. The STM measurements of the HPVD-WSe_2_ were performed at temperature of 10K using commercial closed-cycle Infinity Omicron SPM instrument. The STM measurements of the bulk WSe_2_ were performed at room temperature using four-probe Omicron instrument. All STM images were collected in the constant-current mode using a chemically etched tungsten tip that was prepared by repeated indentation into a clean Au (111) surface and calibrated with respect to the Shockley state of the Au (111) surface. The bias voltage values refer to the sample with respect to the STM tip. Prior to STM measurements, the WSe_2_ /HOPG samples were annealed at 500 K for 2 h under UHV conditions, while ME-WSe_2_ was produced by in situ exfoliation of the bulk WSe_2_ crystals in the UHV conditions. The point *dI/dV* were measured using a lock-in amplifier with a bias modulation voltage of 20 meV at a frequency of 815 Hz. The number of defects can be counted based on their topographic appearance. For each STM image, the defect density was calculated as the number of observed defects divided by the corresponding scanned area.

### DFT Simulations

The DFT calculations were performed using the Vienna Ab initio Simulation Package (VASP) [ ^55–57^ with the projector augmented wave (PAW)^58,59^ method and the Perdew–Burke–Ernzerhof (PBE)^60^ exchange-correlation functional. A k-point mesh of 2 × 2 × 1 was used for the Brillouin zone integration, and the cutoff energy for the plane-wave basis was set to 500 eV. To account for the long-range van der Waals (vdW) interactions, which are crucial for accurately describing structural properties of layered materials like WSe_2_, we employed the DFT-D3 dispersion correction [ ^61–63^ method in our DFT calculations. The electronic self-consistent field iterations were converged with a strict energy tolerance of 1 × 10^–6^ eV. For ionic relaxations, the convergence criterion was set to a maximum Hellmann–Feynman force of 0.02 eV/Å on each atom. A vacuum layer of 15 Å was introduced along the *z*-direction to minimize the interaction between periodic images of the doped WSe_2_ monolayers. The atomic structure models of the doped WSe_2_ monolayers were visualized using the VESTA software package,^64^ a tool for visualizing three-dimensional structural models based on crystallographic data. The preparation of input files for DFT calculations, as well as the analysis and visualization of the computed results, were performed using the VASPKIT program.^65^

Synopsys QuantumATK T-2022.03 ^66^ was utilized to calculate phonon-limited mobility, which could be divided into the following three steps. First, geometry optimization is performed with force tolerance of 0.02 eV Å^–1^. Second, relaxed structures take 3 × 3 × 1 repetitions, and dynamical matrix of supercells is calculated. Third, the derivative of the Hamiltonian is calculated, the one and dynamical matrix are employed to calculate electron–phonon coupling matrix. Then, mobility could be extracted after electron–phonon coupling calculations. The configurations of linear Combination of Atomic Orbitals (LCAO) calculator include: Perdew–Burke–Ernzerhof generalized gradient approximation (PBE-GGA) exchange-correlation functional; Hartwigsen-Goedecker-Hutter (HGH) pseudopotentials with Tier4 basis sets; 240 Ry mesh cutoff; 8 × 8 × 1 k-point sampling; DFT-D3 dispersion correction; 1 × 10^–6^ eV convergence tolerance of self-consistent iteration loop.

### FET Fabrication and Electrical Performance Measurement

The WSe_2_ films were transferred by the poly­(methyl methacrylate) (PMMA)-assisted wet transfer method. First, PMMA was spin-coated onto the monolayer WSe_2_ films. After soaking in deionized water, the free-standing PMMA/WSe_2_ stacks were delaminated from substrates and transferred to P^2+^ Si substrates with a 270 nm SiO_2_ layer. For CVD-WSe_2_ FETs, the monolayer CVD-WSe_2_ film was transferred to P^2+^ Si substrates with an 8 nm atomic layer deposition (ALD) deposited HfO_2_ layer. To dry the samples and enhance adhesion, the PMMA/WSe_2_ stacks were postbaked on the hot plate at 120 °C for 20 min under vacuum. To remove PMMA residue, PMMA/WSe_2_ stacks were soaked in 80 °C hot acetone for 30 min. Monolayer VPVD-WSe_2_, CPVD-WSe_2_, HPVD-WSe_2_, and CVD-WSe_2_ transistors were fabricated by electron beam lithography (EBL). O_2_ (10 mTorr, 100 W) reactive ion gas plasma was applied to define the channel region and source/drain area pattern, followed by electron beam evaporation of 20 nm Pt/20 nm Au and sequent lift off. Electrical performance was measured by Keysight B1500A semiconductor parameter analyzer with a closed-cycle cryogenic probe station and all device measurements were performed under a vacuum environment (∼10^–6^ Torr). The mobilities of the monolayer WSe_2_ in Figure 4 were calculated by the equation: μ = (d*I*_*d*_/d*V*_*g*s_) × [L_ch_/(WC_ox_ *V*_*ds*_)], where *I*_*d*_ is the drain current, *V*_*g*s_ is the gate voltage, L_ch_ is the channel length, W is the channel width, C_ox_ is the capacitance per unit area of the gate dielectric (12.7 nFcm^–2^ for 270 nm SiO_2_), and *V*_*ds*_ is the voltage between the source/drain.

## Supplementary Material



## Notes

DFT calculations of hole mobility and band structure for various defects, OM images, AFM images, STEM images, STM images, SEM images of the WSe_2_, XRD of the precursors, Raman and PL of the different types of WSe_2_, XPS of the different types of WSe_2_, Electrical properties of the different types of WSe_2_. (PDF)

## Notes

Notice: This manuscript has been authored by UT-Battelle, LLC, under contract DE-AC05–00OR22725 with the US Department of Energy (DOE). The US government retains and the publisher, by accepting the article for publication, acknowledges that the US government retains a nonexclusive, paid-up, irrevocable, worldwide license to publish or reproduce the published form of this manuscript, or allow others to do so, for US government purposes. DOE will provide public access to these results of federally sponsored research in accordance with the DOE Public Access Plan (http://energy.gov/downloads/doe-public-access-plan).
