# Supplementary material for: Growth of Low-Defect WSe2 Film via High-Purity van der Waals Crystal Precursor
Source: ACS Nano. 2026 Mar 16;20(12):9961–9. doi: 10.1021/acsnano.5c21076 (PMC13045340; doi:10.1021/acsnano.5c21076)
Supplement: Supplementary file 1 [file nn5c21076_si_001.pdf]

Supplementary Materials for

Growth of low-defect WSe_2 film via high-purity van der Waals crystal precursor

Hang Liu *et al*.

*Corresponding authors: Kian Ping Loh (kian-ping.loh@polyu.edu.hk), Lain-Jong Li (lanceli1@hku.hk), Xu Lu (xu.lu@kaust.edu.sa)

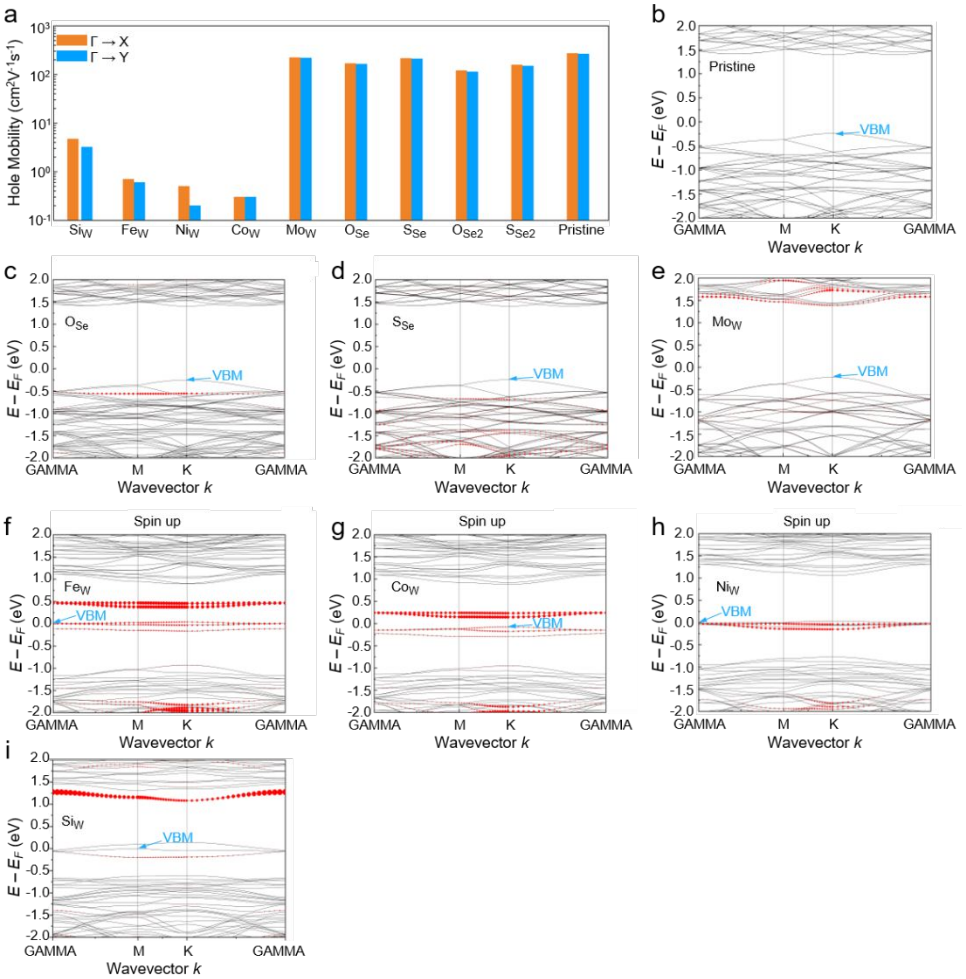

Supplementary Figure 1. DFT calculations of hole mobility and band structure for various defects. (a) Comparison of DFT calculated hole mobilities for pristine, O_{Se}, O_{Se2}, S_{Se2}, S_{Se}, Mo_W, Fe_W, Co_W, Si_W and Ni_W incorporated WSe_2. Band structures of WSe_2 incorporated with (b) pristine, (c) O_{Se}, (d) S_{Se}, (e) Mo_W, (f) Fe_W, (g) Co_W, (h) Ni_W and (i) Si_W defects.

We performed DFT calculations to assess the hole mobility of various cases based on deformation potential theory without considering charge scattering processes. This approach provides preliminary approximation to understand the effect of impurities. The pristine WSe_2 and those incorporated with O_{Se}, O-substituted double sides (top and bottom) Se (O_{Se2}), S-substituted single side Se (S_{Se}), S-substituted double sides (top and bottom) Se (S_{Se2}) and Im_W were calculated, where Im_W includes Mo- (Mo_W), Fe- (Fe_W), Co- (Co_W), Si- (Si_W) and Ni- (Ni_W) substituted W defects. The models were also considered to better match the experimentally measured impurity concentrations. The hole mobility of pristine WSe_2 was calculated to be 264 - 271 $cm^2V^{-1}s^{-1}$

(Supplementary Figure 1a, b and Supplementary Table 1), and O_{Se}, O_{Se2}, $S_{Se,}$ S_{Se2} and Mo_W defects exert minimal effect on hole mobility (113 - 222 $cm^2V^{-1}s^{-1}$) (Supplementary Figure 1c-e). The hole mobilities counted along the $\Gamma \rightarrow X$ and $\Gamma \rightarrow Y$ directions ranged between 0.2 and 0.7 $cm^2V^{-1}s^{-1}$ in Fe_W, Co_W, and Ni_W incorporated WSe_2, three orders of magnitude lower than that of the pristine WSe_2. The hole mobilities in Si_W incorporated WSe_2 counted along the $\Gamma \rightarrow X$ and $\Gamma \rightarrow Y$ directions range from 4.7 to 3.2 $cm^2V^{-1}s^{-1}$, two orders of magnitude lower than that of the pristine WSe_2. The band structure considering spin up in Supplementary Figures 1f-i showed that in-gap states (indicated as red bands) are introduced by these defects, and the reduced curvature of valence band maximum (VBM, indicated by the blue arrow) incurs significant increase in effective masses of holes and thus reduces the hole mobility.

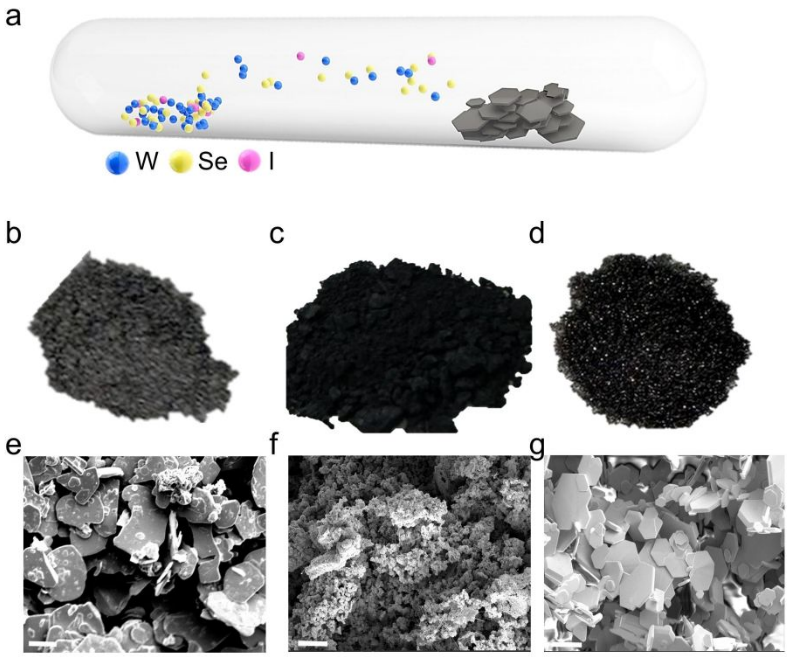

Supplementary Figure 2. Characterizations of different WSe_2 sources. (a) Illustration of CVT synthesis of the WSe_2 bulk vdW crystals. Photographs of (b) commercial, (c) hydrothermal, and (d) CVT-grown vdw crystal precursors. SEM images (scale bars, 10 μm) of (e) commercial powders, (f) hydrothermal powders, and (g) vdW crystals.

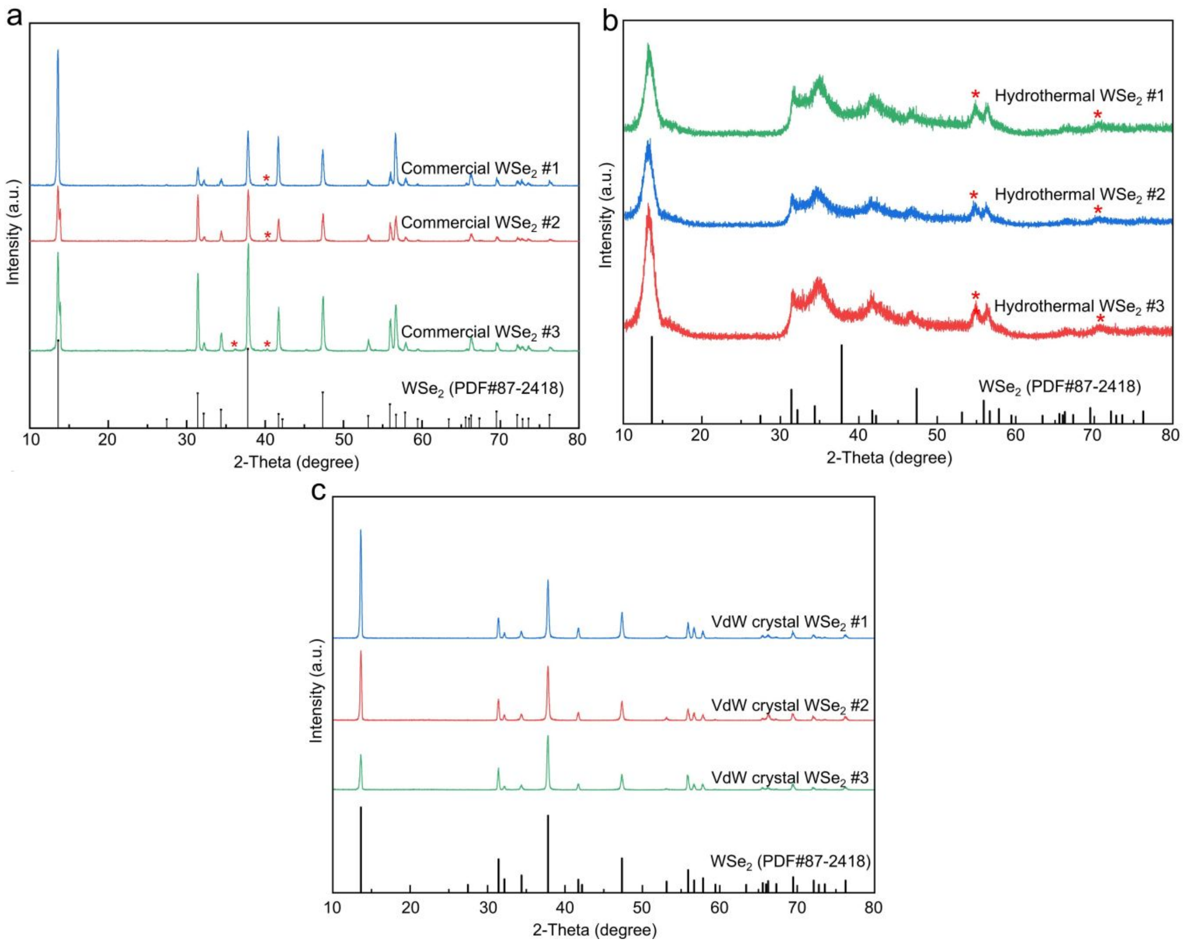

Supplementary Figure 3. Additional XRD spectra of (a) commercial, (b)hydrothermal and (c) vdW crystal WSe_2 precursors through multiple samplings.

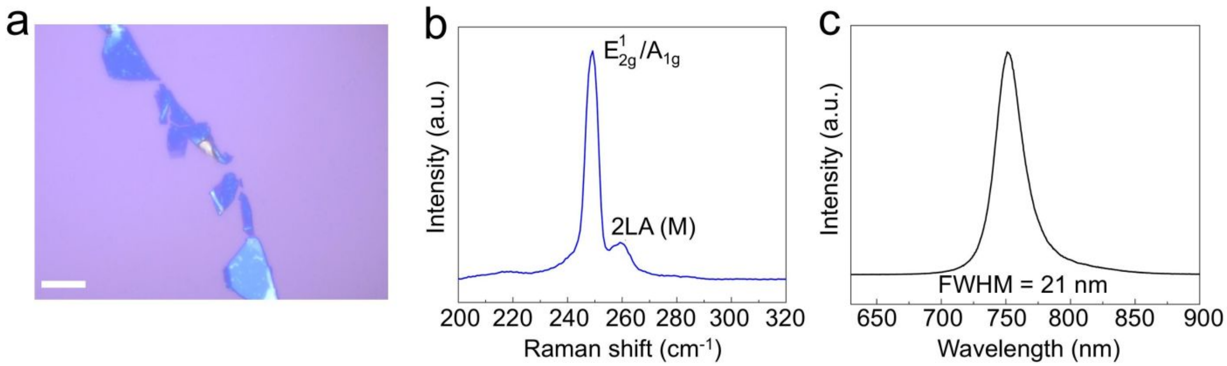

Supplementary Figure 4. Mechanical exfoliation of the CVT-grown vdW crystals. (a) Optical image (scale bar, 10 μm), (b) Raman spectrum, and (c) PL spectrum of the ME-WSe_2 flakes.

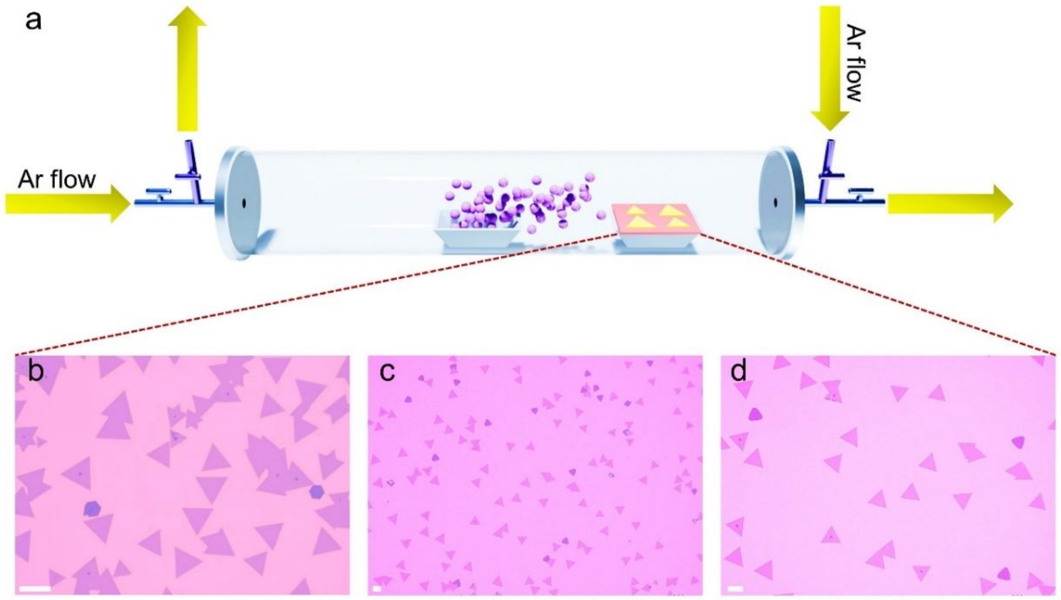

Supplementary Figure 5. Schematics of the reverse-flow PVD system and single-crystal WSe_2 monolayers grown on SiO_2/Si. (a) Illustration of the PVD setup, with the solid WSe_2 powders as the precursor. A backward flow from the substrate to the precursor was utilized to prevent the accidental supply of the reactant vapor during the heating stage. Optical images (scale bars, 50 μm) of the WSe_2 monolayers grown from (b) commercial, (c) hydrothermal, and (d) vdw crystal precursors.

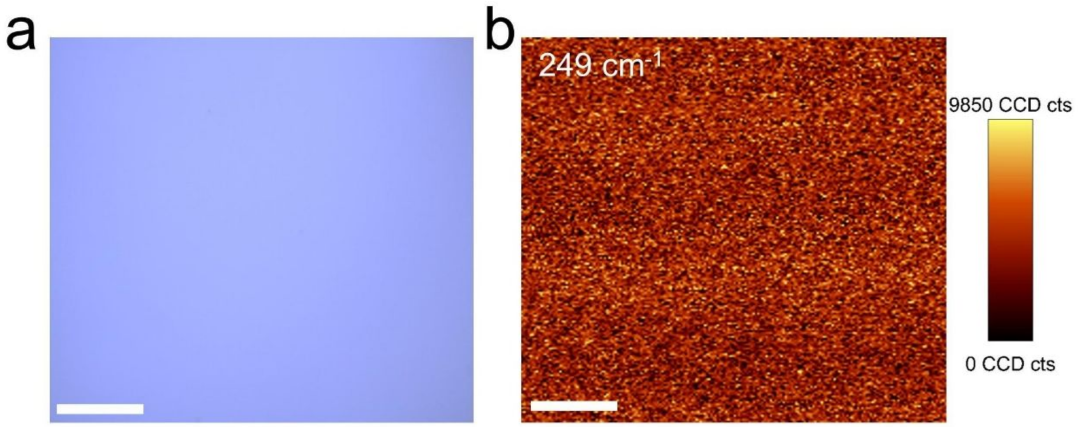

Supplementary Figure 6. Characterizations of VPVD-WSe_2 film. (a) Supplementary optical image and (b) Raman mapping of the VPVD-WSe_2 monolayer film (scale bar, 20 μm).

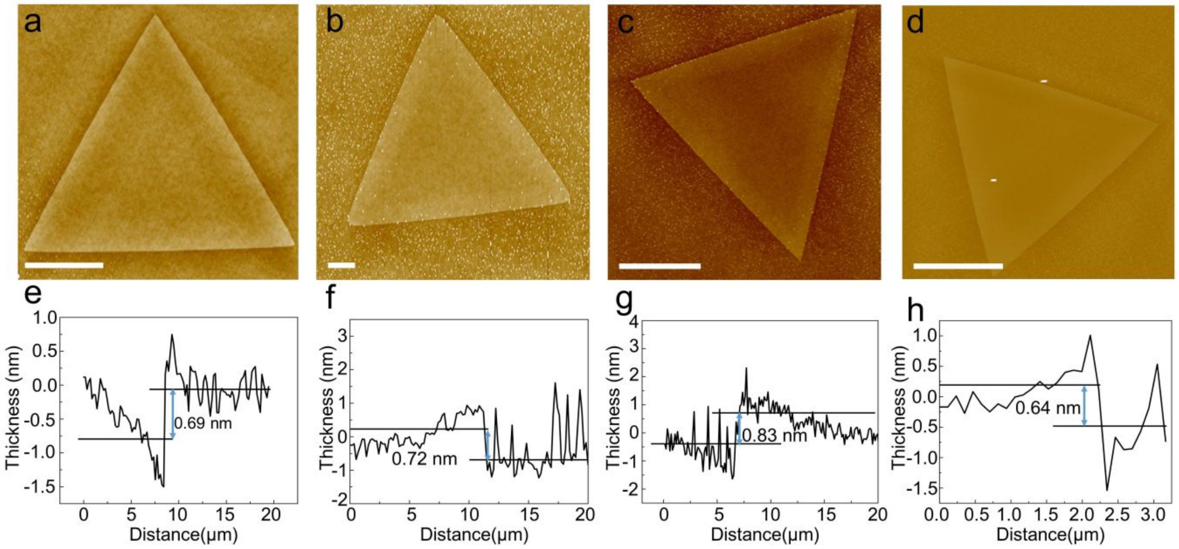

Supplementary Figure 7. AFM of WSe_2 monolayers using different sources. (a-d) AFM images of WSe_2 single crystals synthesized from commercial, hydrothermal, vdW crystal precursors and CVD, respectively (scale bars, 10 μm). (e-h) Thickness profiles corresponding to a, b, c and d, respectively.

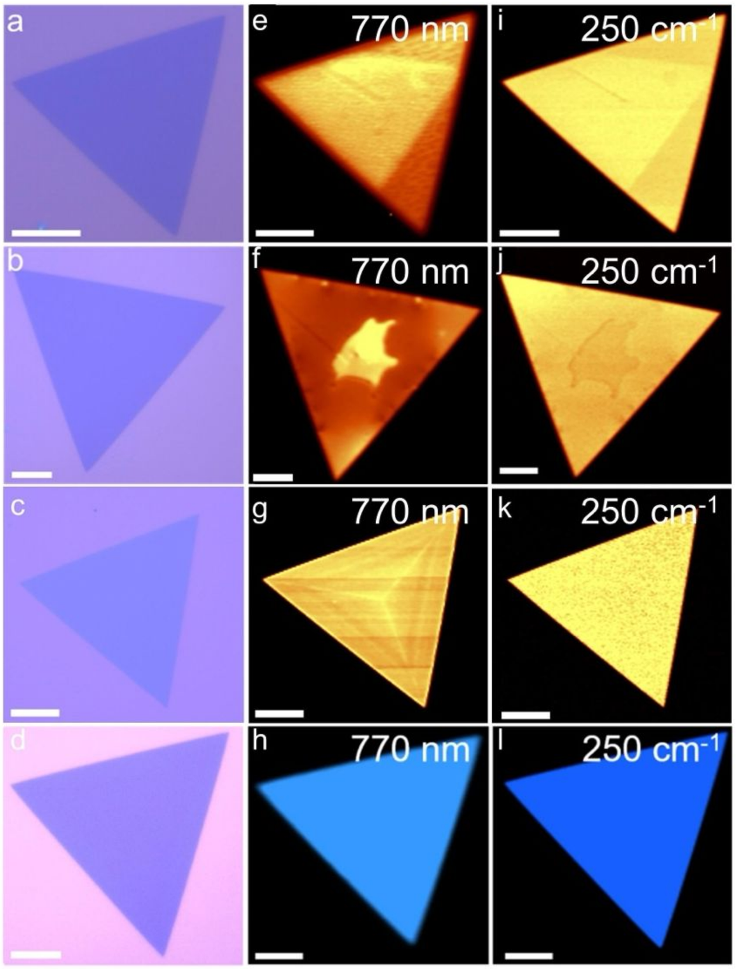

Supplementary Figure 8. Characterization of WSe_2 monolayers. (a-d) Optical microscopy images of (a) CPVD-WSe_2, (b) HPVD-WSe_2, (c) CVD-WSe_2 and (d) VPVD-WSe_2 monolayers. (e-h) PL mapping of (e) CPVD-WSe_2, (f) HPVD-WSe_2, (g) CVD-WSe_2 and (h) VPVD-WSe_2 monolayers. (i-l) Raman mapping of (i) CPVD-WSe_2, (j) HPVD-WSe_2, (k) CVD-WSe_2 and (l) VPVD-WSe_2 monolayers. Scale bars, 10 μm.

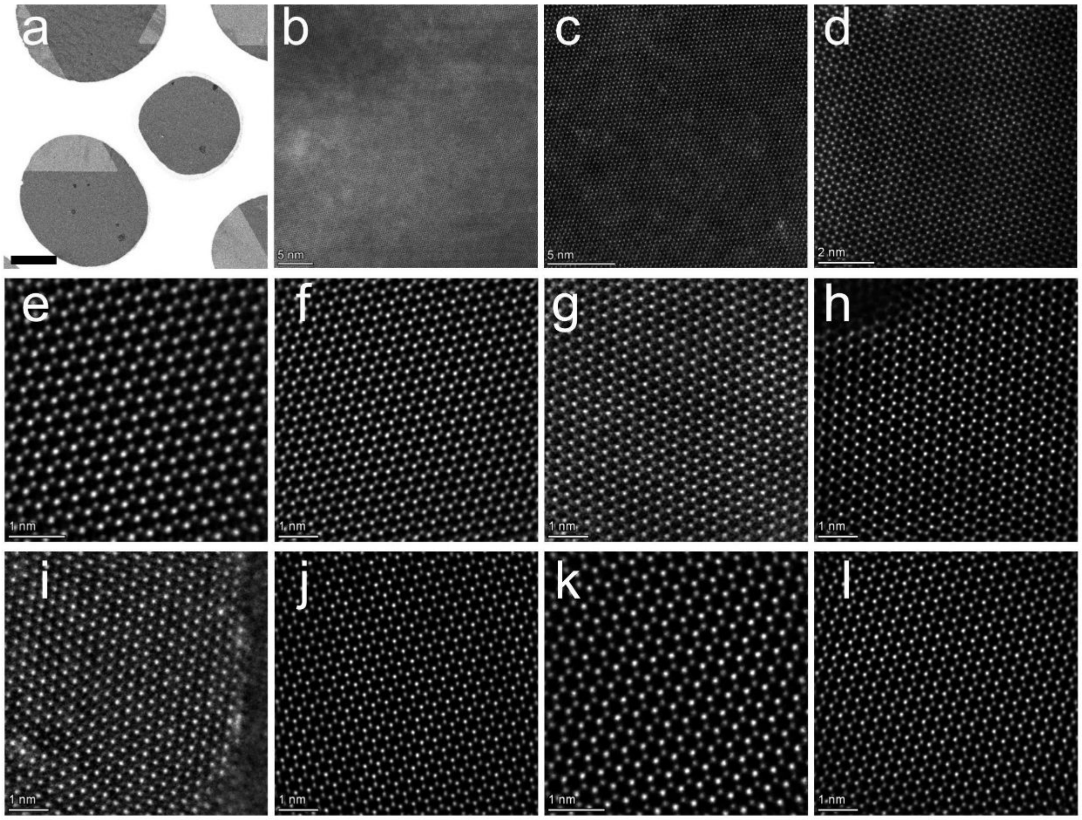

Supplementary Figure 9. STEM images collected at different regions on different VPVD-WSe_2 monolayers. (a) Bright-field TEM image of multiple VPVD-WSe_2 monolayers after being transferred on Cu grid (scale bar, 10 μm). (b-l) STEM images of VPVD-WSe_2, displaying flawless hexagonal honeycomb lattice structure.

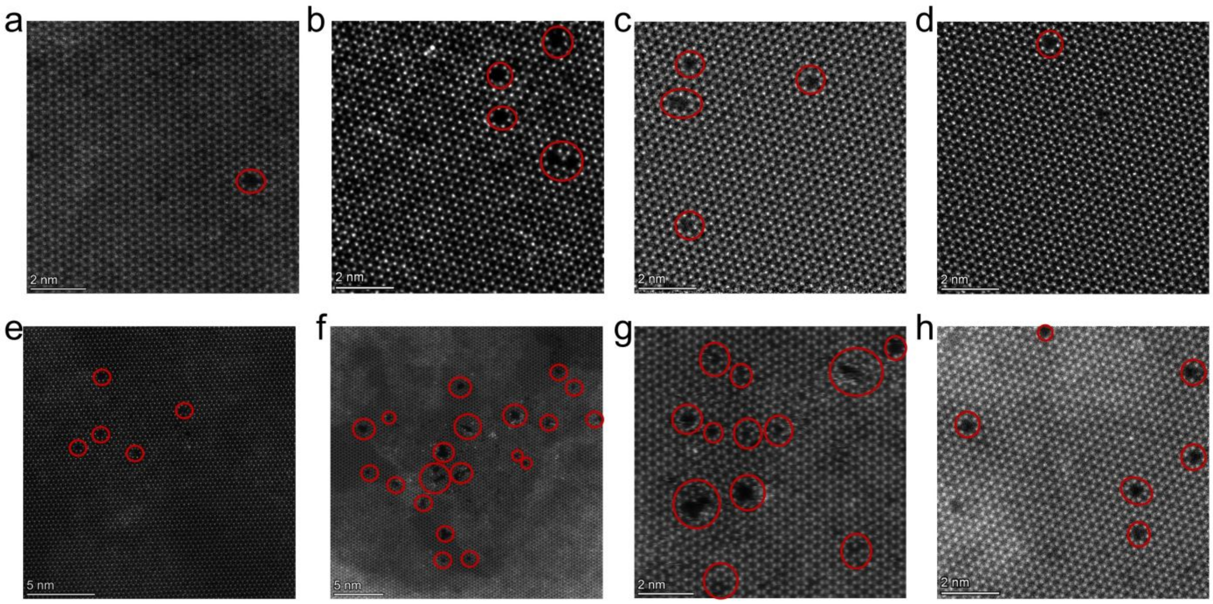

Supplementary Figure 10. STEM images of (a-d) CPVD-WSe_2 and (e-h) HPVD-WSe_2. The low-contrast holes marked by red circles suggested the occasional cluster defects.

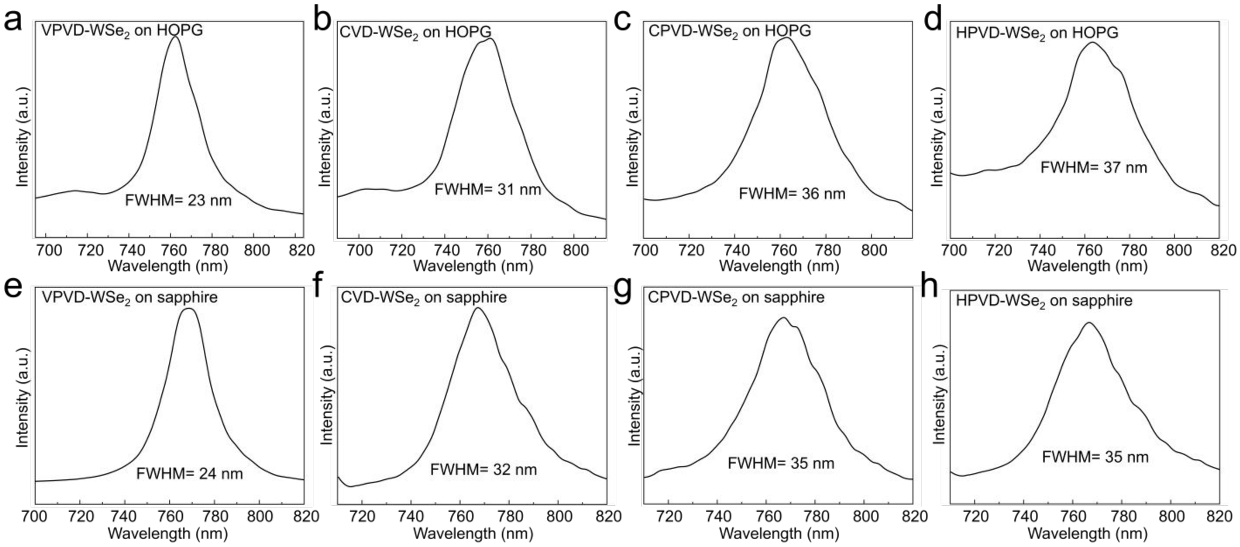

Supplementary Figure 11. (a-d) Room temperature PL spectra of WSe_2 grown on HOPG. (e-h) Room temperature PL spectra spectra of WSe_2 grown on sapphire.

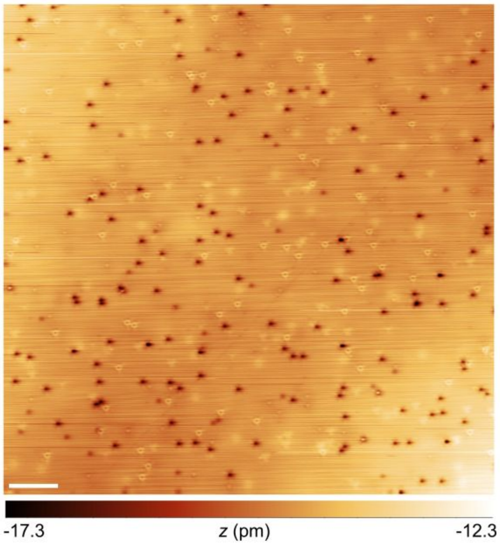

Supplementary Figure 12. STM image (scale bar, 5 μm) of HPVD-WSe_2 (V = 1.5 V, I = 90 pA).

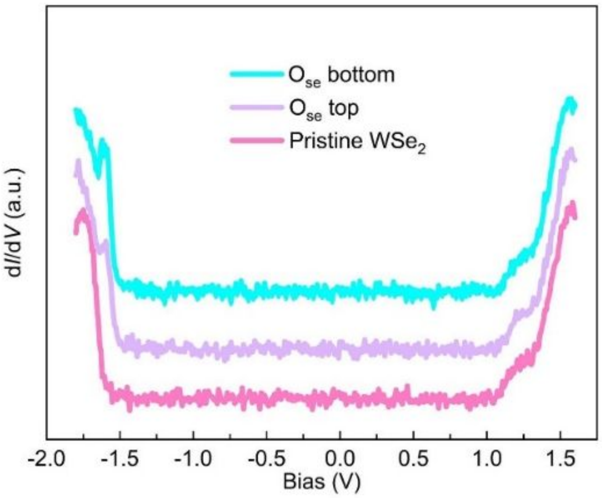

Supplementary Figure 13. dI/dV spectra of the top (aqua) and bottom (lavender) O_{se}, and pristine WSe_2 (pink). The O_{se} defects exhibited a bandgap similar to pristine WSe_2, with a distinct defect resonance situated deep inside the valence band.

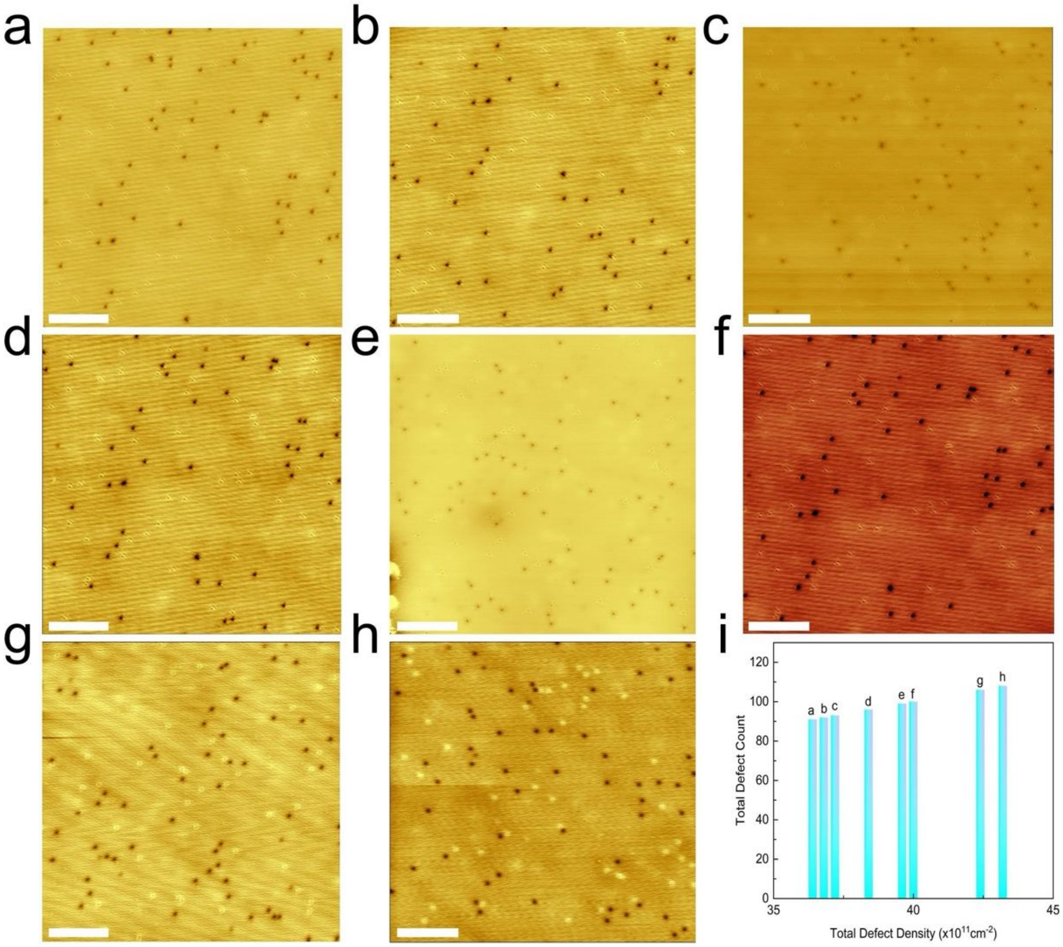

Supplementary Figure 14. STM of the VPVD-WSe_2. (a-h) STM images of randomly selected VPVD-WSe_2 monolayers (scale bar, 10 nm). (i) Statistics of total defect densities corresponding to a-h.

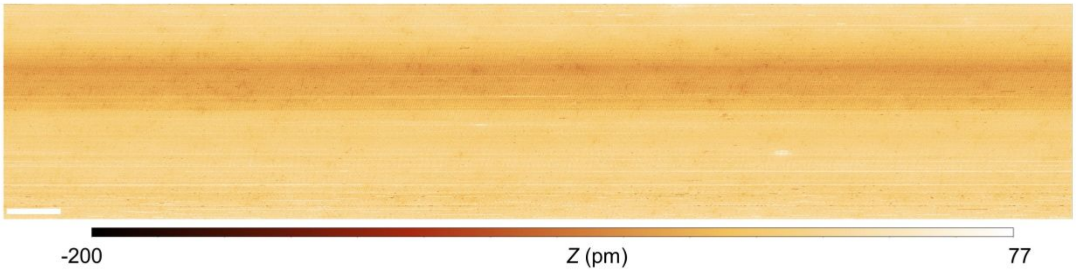

Supplementary Figure 15. STM image (scale bar, 10 μm) of WSe_2 mechanically exfoliated from our bulk crystals (V = -1.75 V, I = 40 pA).

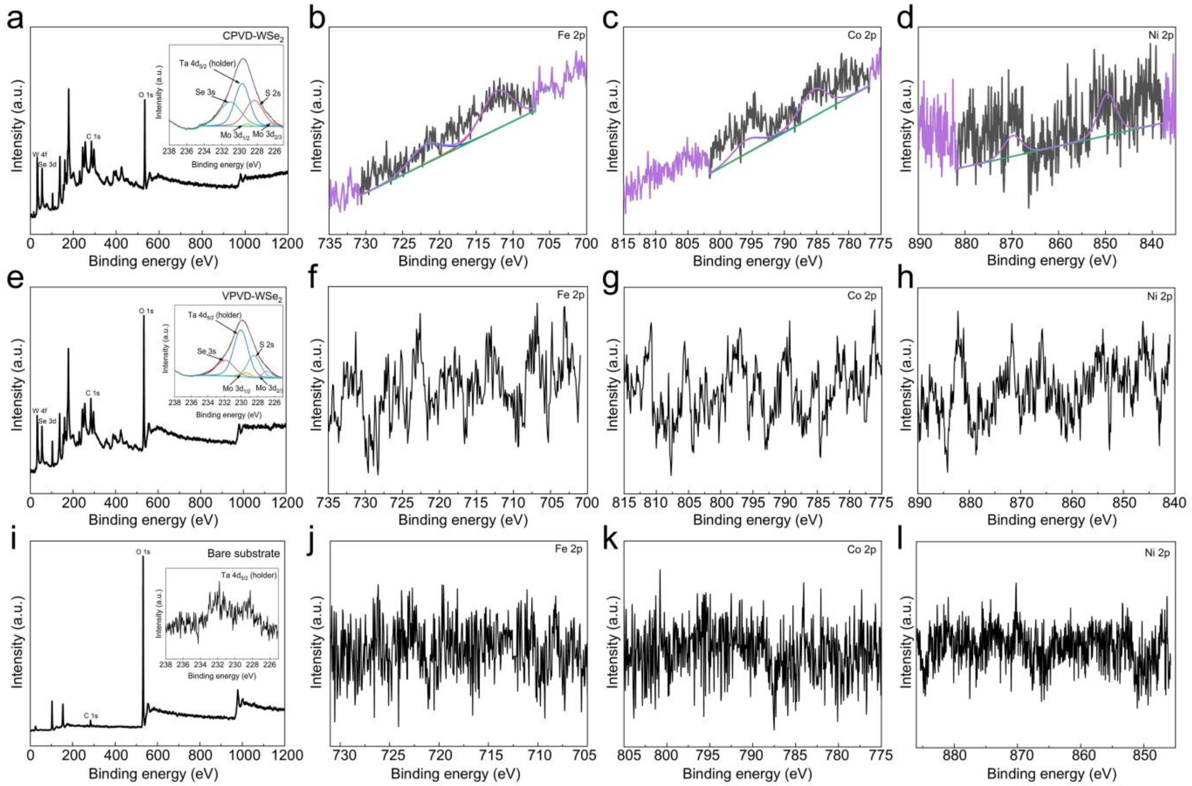

Supplementary Figure 16. High-resolution XPS spectra of WSe_2. (a) Full-scan XPS spectrum of CPVD-WSe_2. (b) Fe 2p, (c) Co 2p, and (d) Ni 2p XPS spectra of CPVD-WSe_2. (e) Full-scan XPS spectrum of VPVD-WSe_2. (f) Fe 2p, (g) Co 2p, and (h) Ni 2p XPS spectra of VPVD-WSe_2. (i) Full-scan XPS spectrum of bare substrate. (j) Fe 2p, (k) Co 2p, and (l) Ni 2p XPS spectra of bare substrate.

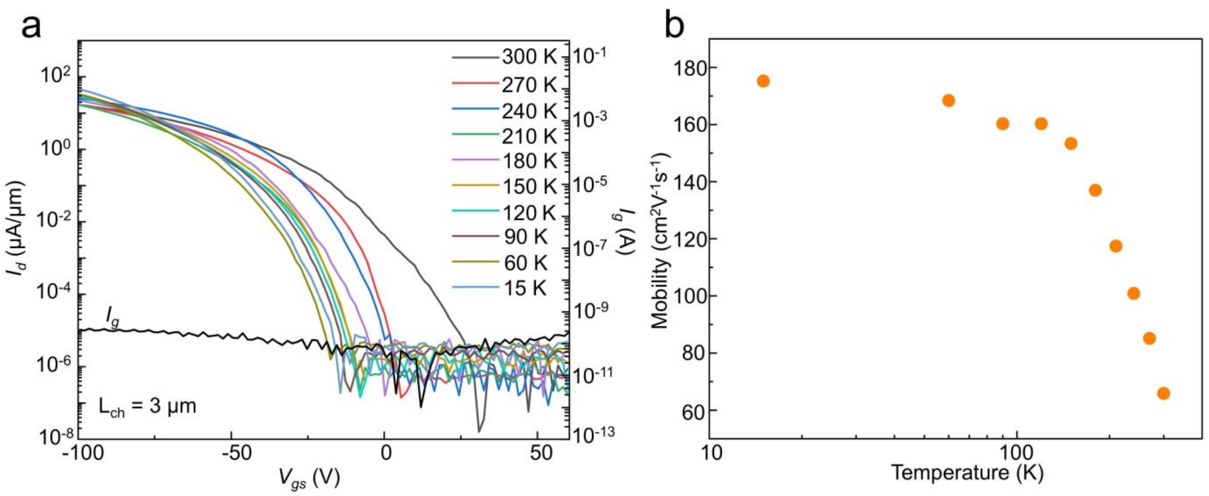

Supplementary Figure 17. (a) Transfer curves of a typical VPVD-WSe_2 measured at different temperatures at V_{ds} = -1 V. (b) Field-effect hole mobility as a function of temperature for VPVD-WSe_2.

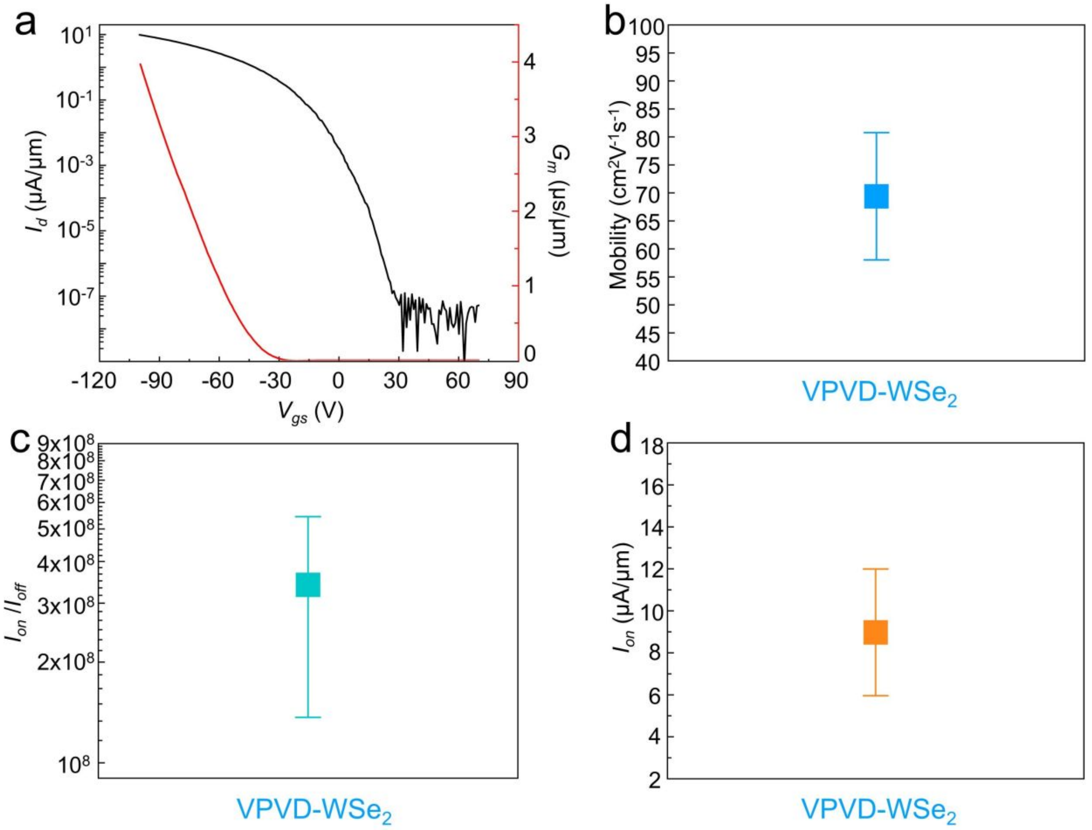

Supplementary Figure 18. Transconductance as a function of gate voltage of a typical VPVD-WSe_2 FET at V_{ds} = -1 V. Error bars in (b) mobility, (c) on/off ratio and (d) on current of the measured VPVD-WSe_2 devices.

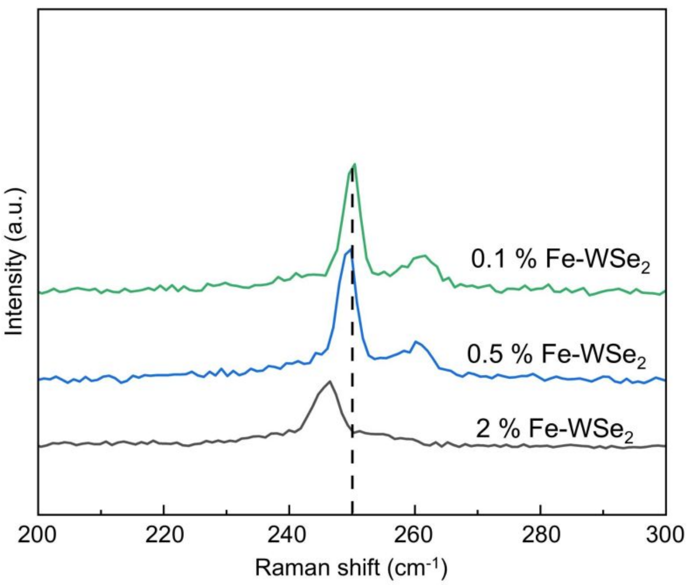

Supplementary Figure 19. Raman spectra of 0.1 ,0.5, and 2% Fe doped WSe_2.

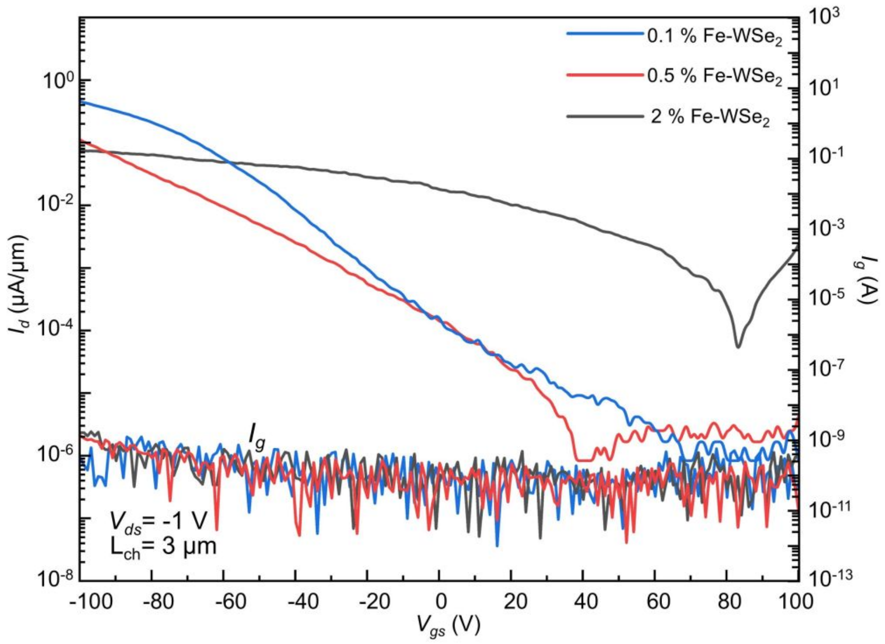

Supplementary Figure 20. Transfer curves of the VPVD-WSe_2 with different doping concentration of Fe.

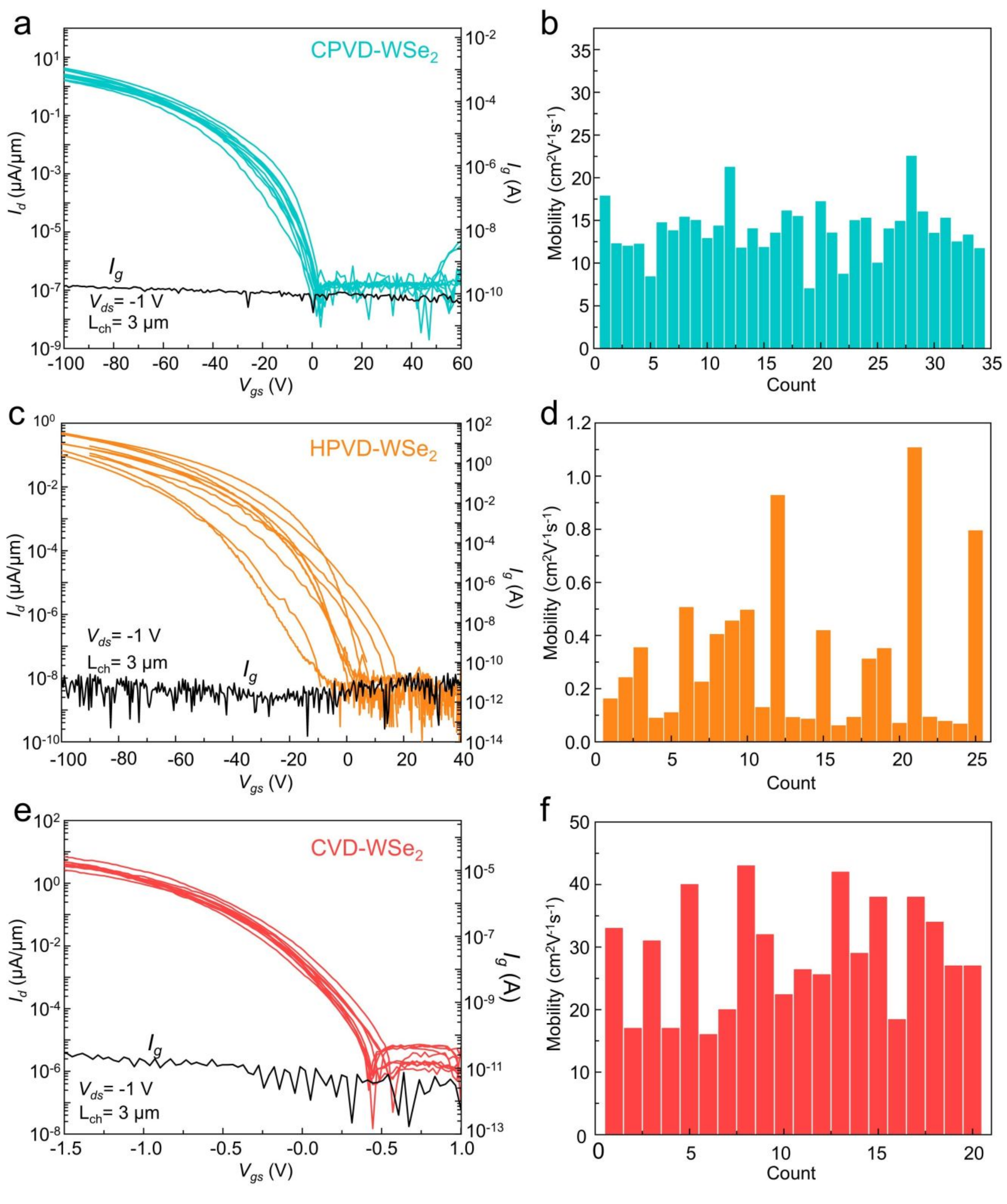

Supplementary Figure 21. FET transfer characteristics of (a) CPVD-WSe_2 (c) HPVD-WSe_2 and (e) CVD-WSe_2 at V_{ds} = -1 V. Statistical distribution of hole μ_{FE} for the (b) CPVD-WSe_2 and (d) HPVD-WSe_2 and (f) CVD-WSe_2 based FETs.

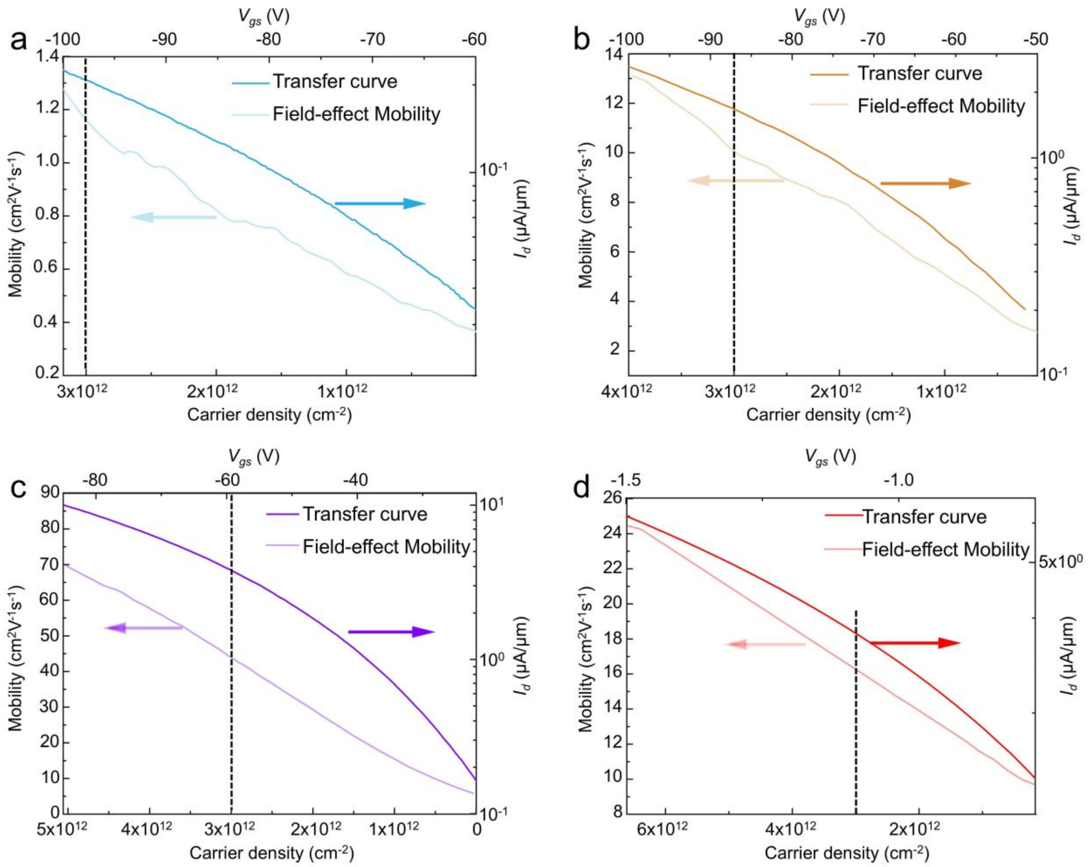

Supplementary Figure 22. Mobility changes as a function of carrier concentration for (a) CPVD-WSe_2, (b) HPVD-WSe_2, (c) VPVD-WSe_2 and (d) CVD-WSe_2 FET devices.

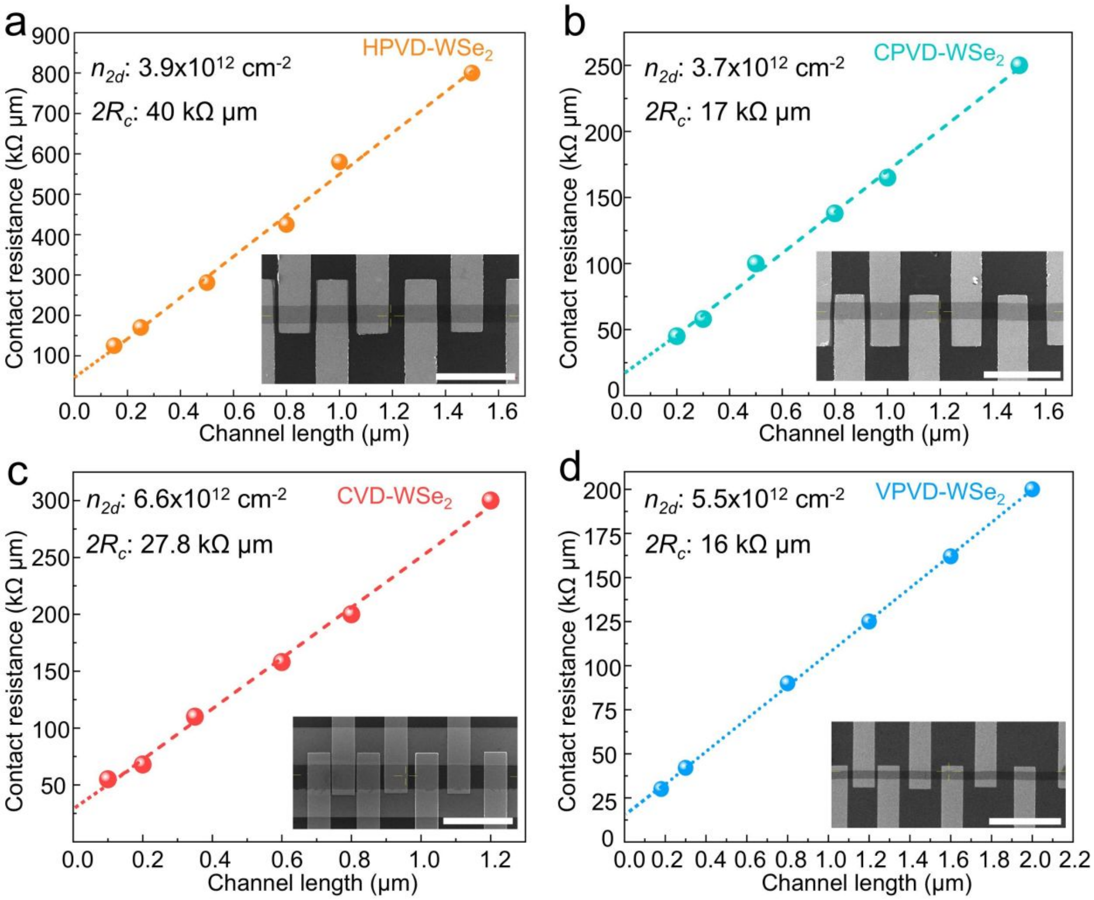

Supplementary Figure 23. The contact resistance values extracted for (a) HPVD-WSe_2, (b) CPVD-WSe_2, (c) CVD-WSe_2 and (d) VPVD-WSe_2 devices using the transfer-length method. Inset, false color SEM images of the TLM structure. Scale bars, 5 μm.

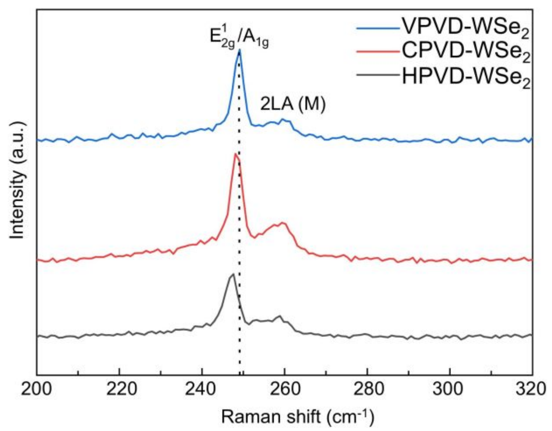

Supplementary Figure 24. Raman spectra of HPVD-WSe_2, CPVD-WSe_2 and VPVD-WSe_2 after device fabrication.

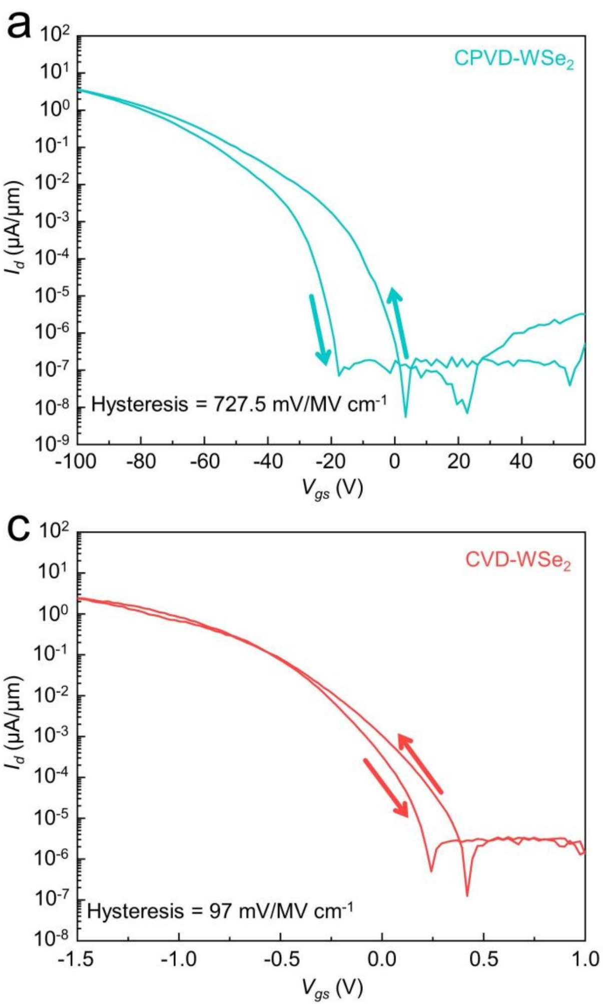

Supplementary Figure 25. Typical dual-sweep transfer curve characteristics of (a) CPVD-WSe_2, (b) HPVD-WSe_2, (c) CVD-WSe_2 and (d) VPVD-WSe_2 FETs.

Supplementary Table 1. Calculated hole mobilites for pristine WSe_2 and WSe_2 incorporated with different substitutional defects.

Materials, k-path	Hole mobility, μ_{2D}, $cm^2s^{-1}V^{-1}$
Fe_W, $\Gamma \rightarrow X$	0.7
Fe_W, $\Gamma \rightarrow Y$	0.6
Ni_W, $\Gamma \rightarrow X$	0.5
Ni_W, $\Gamma \rightarrow Y$	0.2
Co_W, $\Gamma \rightarrow X$	0.3
Co_W, $\Gamma \rightarrow Y$	0.3
Si_W, $\Gamma \rightarrow X$	4.7
Si_W, $\Gamma \rightarrow Y$	3.2
Mo_W, $\Gamma \rightarrow X$	222
Mo_W, $\Gamma \rightarrow Y$	218
O_{Se}, $\Gamma \rightarrow X$	168
O_{Se}, $\Gamma \rightarrow Y$	163
S_{Se}, $\Gamma \rightarrow X$	214
S_{Se}, $\Gamma \rightarrow Y$	209
O_{Se2}, $\Gamma \rightarrow X$	120
O_{Se2}, $\Gamma \rightarrow Y$	113
S_{Se2}, $\Gamma \rightarrow X$	157
S_{Se2}, $\Gamma \rightarrow Y$	149
Pristine, $\Gamma \rightarrow X$	271
Pristine, $\Gamma \rightarrow Y$	264

Supplementary Table 2. Histograms table of observed point defect number in different types of WSe_2.

Method	Total defect number	O_{se} top number	O_{se} bottom number	Im_W number
HPVD	335	110	85	140
CPVD	1243	700	543	103
CVD	184	115	49	20
VPVD	91	45	44	2

For each STM image, the defect density was calculated as the number of observed defects divided by the corresponding scanned area. For HPVD-WSe_2, the defect density of $O_{Se(top)}$ is 110/(50 nm × 50 nm) = 4.4 × 10^{12} cm^{-2}. The defect density of $O_{Se(bottom)}$ is 85/(50 nm × 50 nm) = 3.4 × 10^{12} cm^{-2}. The defect density of Im_W is140/(50 nm × 50 nm) = 5.6 × 10^{12} cm^{-2}. For CPVD-WSe_2, the defect density of $O_{Se(top)}$ is 700/(50 nm × 50 nm) = 2.8 × 10^{13} cm^{-2}. The defect density of $O_{Se(bottom)}$ is 543/(50 nm × 50 nm) = 2.17 × 10^{13} cm^{-2}. The defect density of Im_W is 103/(50 nm × 50 nm) = 4.12 × 10^{12} cm^{-2}. For CVD-WSe_2, the defect density of $O_{Se(top)}$ is 115/50 nm × 50 nm = 4.6 × 10^{12} cm^{-2}. The defect density of $O_{Se(bottom)}$ is 49/(50 nm × 50 nm) = 1.96 × 10^{12} cm^{-2}. The defect density of Im_W is 20/(50 nm × 50 nm) = 8 × 10^{11} cm^{-2}. For VCVD-WSe_2, the defect density of $O_{Se(top)}$ is 45/50 nm × 50 nm = 1.8 × 10^{12} cm^{-2}. The defect density of $O_{Se(bottom)}$ is 49/(50 nm × 50 nm) = 1.76 × 10^{12} cm^{-2}. The defect density of Im_W is 2/(50 nm × 50 nm) = 8 × 10^{10} cm^{-2}.

Supplementary Table 3. Benchmark table for WSe_2 based FETs.

2D materials	Gate stack	S/D contacts	Channel length (μm)	I_{on} (μA/μm)	μ_{FE} ($cm^2V^{-1}s^{-1}$)	I_{on}/I_{off}	Ref.
Poly-crystalline VPVD monolayer WSe_2 film	270 nm SiO_2	Pt/Au	3	21 (V_{ds}= -1V)	112	$>10^8$	This work
Poly-crystalline CPVD monolayer WSe_2 film	270 nm SiO_2	Pt/Au	3	1 (V_{ds}= -1V)	14	$>10^8$	This work
Poly-crystalline HPVD monolayer WSe_2 film	270 nm SiO_2	Pt/Au	3	0.03 (V_{ds} = -1V)	0.31	$>10^7$	This work
Poly-crystalline CVD monolayer WSe_2 film	8 nm HfO_2	Pt/Au	3	3 (V_{ds}= -1V)	29	$>10^6$	This work
Single-crystalline PVD bilayer WSe_2 flake	285 nm SiO_2	VdW contact	2	0.01 (V_{ds} = 1V)	10	10^4	(*1*)
Single-crystalline PVD monolayer WSe_2 flake	285 nm SiO_2	VdW contact	4	10 (V_{ds} = 1V)	90	10^5	(*2*)
Single-crystalline PVD monolayer WSe_2 flake	285 nm SiO_2	VdW contact	5	21 (V_{ds} = 1V)	92	$>10^7$	(*3*)
Single-crystalline PVD monolayer WSe_2 flake	285 nm SiO_2	Cr/Au	Not verified	Not verified	7	10^7	(*4*)

Single-crystalline monolayer CVD WSe_2 film	300 nm SiO_2	Pt/Au	0.7	108.1 (V_{ds} = –1V)	72.8	10^8	(*5*)
Single-crystalline CVD monolayer WSe_2 flake	50 nm Al_2O_3	Ni/Au	1	0.7 (V_{ds} = 1V)	1	10^6	(*6*)
Poly-crystalline CVD trilayer WSe_2 film	300 nm SiO_2	Pd	Not verified	60 (V_{ds} = 0.1V)	2.2	10^6	(*7*)
Poly-crystalline CVD monolayer WSe_2 film	h-BN	Ti/Pd	1	11 (V_{ds} = 1V)	83	10^7	(*8*)
Single-crystalline CVD monolayer WSe_2 film	100 nm SiO_2	Ni/Au	1	1 (V_{ds}= -0.2V)	4.2	10^7	(*9*)
Poly-crystalline CVD bilayer WSe_2 film	PEO: $CsClO_4$	Cr/Pd/ Au	10	1-10 (V_{ds}= 0.5V)	30	10^7	(*10*)
Single-crystalline CVD bilayer WSe_2 flake	20 nm HfO_2	Ni/Au	1	42 (V_{ds}= 1V)	22-32	$>10^8$	(*11*)
Single-crystalline monolayer WSe_2 flake	300 nm SiO_2	Cr/Pd/ Au	2.75	30 (V_{ds}= 1V)	102	10^7	(*12*)
Single-crystalline monolayer WSe_2 flake	Si_3N_4/SiO_2 (100:300 nm)	Pd	10	Not verified	100	10^6	(*13*)
Poly-crystalline CVD	285 nm SiO_2	Ti/Au	Not verified	17 (V_{ds}= -1V)	21	$>10^5$	(*14*)

monolayer WSe_2 film							
Single-crystalline CVD monolayer WSe_2 flake	300 nm SiO_2	Au	1	100 (V_{ds}= 2V)	100	10^8	(*15*)
Poly-crystalline CVD monolayer WSe_2 film	PS-PMMA-PS	Ni/Au	7	10 (V_{ds}= –0.1V)	90	10^5	(*16*)
Single-crystalline CVD monolayer WSe_2 flake	Ionic liquid	Pd/Au	Not verified	50 (V_{ds}= -0.5V)	11	10^6	(*17*)
Poly-crystalline CVD bilayer WSe_2 film	Not verified	Cr/Au	Not verified	Not verified	5.3	10^4	(*18*)
Poly-crystalline CVD monolayer WSe_2 film	PS-PMMA-PS	Ni/Au	Not verified	Not verified	55	10^5	(*19*)
Single-crystalline CVD monolayer WSe_2 flake	6 nm HfO_2	Pt	0.018	727 (V_{ds}= -1.5V)	Not verified	10^9	(*20*)
Single-crystalline CVD monolayer WSe_2 flake	2.8 nm HfO_2	Ti/Pd/Au	0.065	300 (V_{ds}= -1V)	35	10^6	(*21*)
Single-crystalline CVD bilayer WSe_2 flake	6 nm SiO_2	Pt/Au	0.12	425 (V_{ds}= -1V)	90	10^8	(*22*)
Single-crystalline CVD	12 nm HfO_2	Ni/Pd/Au	0.135	594 (V_{ds}= -1V)	Not verified	10^7	(*23*)

bilayer WSe_2 flake							
Poly-crystalline CVD monolayer WSe_2 film	AlO_x/ HfO_2	Ru	0.1	50 (V_{ds}= 1V)	Not verified	10^7	(*24*)
Exfoliated monolayer WSe_2 flake	300 nm SiO_2	Pt/Au	0.8	110.3 (V_{ds}= -1V)	84.3	10^8	(*5*)
Exfoliated WSe_2 flake (12L, 8 nm)	270 nm SiO_2	Ti/Au	15.8	0.8 (V_{ds}= 2V)	302	10^6	(*25*)
Exfoliated monolayer WSe_2 flake	270 nm SiO_2	Pd/Au	8	Not verified	140	10^6	(*26*)
Exfoliated monolayer WSe_2 flake	72 nm Al_2O_3	Ag/Au	1	210 (V_{ds}= 3V)	142	10^6	(*27*)
Exfoliated monolayer WSe_2 flake	300 nm SiO_2	Au	6.1	Not verified	200	10^6	(*28*)

References

1. J. Li *et al.*, General synthesis of two-dimensional van der Waals heterostructure arrays. *Nature* **579**, 368-374 (2020).
2. Z. Zhang *et al.*, Ultrafast growth of large single crystals of monolayer WS_2 and WSe_2. *National Science Review* **7**, 737-744 (2020).
3. Z. Zhang *et al.*, Highly Selective Synthesis of Monolayer or Bilayer WSe_2 Single Crystals by Pre-annealing the Solid Precursor. *Chemistry of Materials* **33**, 1307-1313 (2021).
4. H. Ma *et al.*, In-plane epitaxial growth of 2D CoSe-WSe_2 metal-semiconductor lateral heterostructures with improved WSe_2 transistors performance. *InfoMat* **3**, 222-228 (2021).
5. K. S. Kim *et al.*, Non-epitaxial single-crystal 2D material growth by geometric confinement. *Nature* **614**, 88-94 (2023).
6. A. Kozhakhmetov *et al.*, Scalable Substitutional Re-Doping and its Impact on the Optical and Electronic Properties of Tungsten Diselenide. *Advanced Materials* **32**, 2005159 (2020).
7. K. Park *et al.*, Uniform, large-area self-limiting layer synthesis of tungsten diselenide. *2D Materials* **3**, 014004 (2016).
8. B. Liu *et al.*, High-Performance WSe_2 Field-Effect Transistors via Controlled Formation of In-Plane Heterojunctions. *ACS Nano* **10**, 5153-5160 (2016).
9. X. Zhang *et al.*, Defect-Controlled Nucleation and Orientation of WSe_2 on hBN: A Route to Single-Crystal Epitaxial Monolayers. *ACS Nano* **13**, 3341-3352 (2019).
10. Y.-C. Lin *et al.*, Realizing Large-Scale, Electronic-Grade Two-Dimensional Semiconductors. *ACS Nano* **12**, 965-975 (2018).
11. X. Wang *et al.*, High-performance n-type transistors based on CVD-grown large-domain trilayer WSe_2. *APL Materials* **9**, 071109 (2021).
12. S. Li *et al.*, Halide-assisted atmospheric pressure growth of large WSe_2 and WS_2 monolayer crystals. *Applied Materials Today* **1**, 60-66 (2015).
13. J. Chen *et al.*, Chemical Vapor Deposition of Large-Sized Hexagonal WSe_2 Crystals on Dielectric Substrates. *Advanced Materials* **27**, 6722-6727 (2015).
14. M. Chen *et al.*, Gold-vapor-assisted chemical vapor deposition of aligned monolayer WSe_2 with large domain size and fast growth rate. *Nano Research* **13**, 2625-2631 (2020).
15. H. Zhou *et al.*, Large Area Growth and Electrical Properties of p-Type WSe_2 Atomic Layers. *Nano Letters* **15**, 709-713 (2015).
16. J.-K. Huang *et al.*, Large-Area Synthesis of Highly Crystalline WSe_2 Monolayers and Device Applications. *ACS Nano* **8**, 923-930 (2014).
17. A. Han et al., Growth of 2H stacked WSe_2 bilayers on sapphire. *Nanoscale Horizons* **4**, 1434-1442 (2019).
18. S. V. Mandyam *et al.*, Controlled Growth of Large-Area Bilayer Tungsten Diselenides with Lateral P–N Junctions. *ACS Nano* **13**, 10490-10498 (2019).
19. J. Pu *et al.*, Highly Flexible and High-Performance Complementary Inverters of Large-Area Transition Metal Dichalcogenide Monolayers. *Advanced Materials* **28**, 4111-4119 (2016).
20. H. Lan *et al.*, Wafer-scale CVD Monolayer WSe_2 p-FETs with Record-high 727 μA/μm Ion

and 490 μS/ μm gmax via Hybrid Charge Transfer and Molecular Doping, *2023 International Electron Devices Meeting (IEDM)*, pp 1-4 (2023).

21. C. Chiang, *et al.*, Air-Stable P-Doping in Record High-Performance Monolayer WSe_2 Devices. *IEEE Electron Device Letters* **2022,** *43* (2), 319-322.
22. X. Shi, *et al.*, High-Performance Bilayer WSe_2 pFET with Record I_{ds} = 425 μA/μm and G_m = 100 at μS/μm Vds = -1 V By Direct Growth and Fabrication on SiO_2 Substrate, *2022 International Electron Devices Meeting (IEDM)*, pp 7.1.1-7.1.4 (2022).
23. X. Xiong, *et al.*, Top-Gate CVD WSe_2 pFETs with Record-High Id~594 μA/μm, G_m~244 μS/μm and WSe_2/MoS_2 CFET based Half-adder Circuit Using Monolithic 3D Integration, *2022 International Electron Devices Meeting (IEDM)*, pp 20.6.1-20.6.4 (2022).
24. K. Brien, *et al.*,Advancing 2D Monolayer CMOS Through Contact, Channel and Interface Engineering, *2021 IEEE International Electron Devices Meeting (IEDM)*, pp 7.1.1-7.1.4 (2021).
25. N. R. Pradhan *et al.*, Hall and field-effect mobilities in few layered p-WSe_2 field-effect transistors. *Scientific Reports* **5**, 8979 (2015).
26. H. Fang *et al.*, High-Performance Single Layered WSe_2 p-FETs with Chemically Doped Contacts. *Nano Letters* **12**, 3788-3792 (2012).
27. W. Liu *et al.*, Role of Metal Contacts in Designing High-Performance Monolayer n-Type WSe_2 Field Effect Transistors. *Nano Letters* **13**, 1983-1990 (2013).
28. Z. Wu *et al.*, Defects as a factor limiting carrier mobility in WSe_2: A spectroscopic investigation. *Nano Research* **9**, 3622-3631 (2016).
